# Tn*FLXopen*: Markerless Transposons for Functional Fluorescent Fusion Proteins and Protein Interaction Prediction

**DOI:** 10.1128/spectrum.02428-21

**Published:** 2022-05-02

**Authors:** Felix Dempwolff, Daniel B. Kearns

**Affiliations:** a Department of Biology, Indiana University, Bloomington, Indiana, USA; b Center for Synthetic Microbiology (SYNMIKRO), Marburg, Germany; Washington University in St. Louis

**Keywords:** *Bacillus subtilis*, BiFC, FACS, internal fluorescent protein fusion, protein-protein interactions, transposon mutagenesis, FtsZ, MotA, DivIVA

## Abstract

Fluorescence microscopy of cells expressing proteins translationally linked to a fluorophore can be a powerful tool to investigate protein localization dynamics *in vivo*. One major obstacle to reliably analyze biologically relevant localization is the construction of a fusion protein that is both fluorescent and functional. Here, we develop a strategy to construct fluorescent fusions at theoretically any location in the protein by using Tn*FLXopen* random transposon mutagenesis to randomly insert a gene encoding a fluorescent protein. Moreover, insertions within a target gene are enriched by an inducible gene-trap strategy and selection by fluorescence activated cell sorting. Using this approach, we isolate a variety of fluorescent fusions to FtsZ that exhibit ring-like localization and a fusion to the flagellar stator protein that both is functional for supporting motility and localizes as fluorescent puncta. Finally, we further modify Tn*FLXopen* to insert the coding sequence for the C-terminal half of mVenus for use in bimolecular fluorescence complementation (BiFC) and the *in vivo* detection of protein-protein interaction candidates. As proof-of-concept, the DivIVA polar scaffolding protein was fused to the N terminus of mVenus, the C terminus of mVenus was delivered by transposition, and a combination of fluorescence activated cell sorter (FACS) sorting and whole-genome sequencing identified the known self-interaction of DivIVA as well as other possible candidate interactors. We suggest that the FACS selection is a viable alternative to antibiotic selection in transposon mutagenesis that can generate new fluorescent tools for *in vivo* protein characterization.

**IMPORTANCE** Transposon mutagenesis is a powerful tool for random mutagenesis, as insertion of a transposon and accompanying antibiotic resistance cassette often disrupt gene function. Here, we present a series of transposons with fluorescent protein genes which, when integrated in frame, may be selected with a fluorescence activated cell sorter (FACS). An open reading frame runs continuously through the transposon such that fluorescent protein fusions may be inserted theoretically anywhere in the primary sequence and potentially preserve function of the target protein. Finally, the transposons were further modified to randomly insert a partial fluorescent protein compatible with bimolecular fluorescence complementation (BiFC) to identify protein interaction candidates.

## INTRODUCTION

Transposons are mobile genetic elements that insert themselves into DNA and, as such, are powerful mutagens for forward genetic analysis. Each transposon is defined by two characteristic inverted terminal repeat (ITR) sequences and the DNA between them. A separate translocating enzyme, the transposase, recognizes and mobilizes the ITRs and intervening sequences to other locations in the chromosome (1, 2). Different transposases have different levels of insertion specificity, with some favoring particular locations while others are more random (3–6). The *mariner* transposase *Himar* exhibits almost no regional specificity, requiring only a TA-base doublet which is duplicated upon insertion of its cargo (7). The random insertion of transposons typically disrupts the function of the genes in which they are inserted. When used in genetic screens, it is advantageous for the insertion frequency to be rare, as single insertions in the chromosome are preferable to multiple for the purposes of demonstrating linkage between the insertion and phenotype. Moreover, most transposons are engineered to encode a gene cassette conferring resistance to a particular antibiotic such that rare insertion events can be selected and the mutants subsequently screened.

While transposons are most commonly used for insertional mutagenesis, some systems have been deliberately reengineered to simultaneously introduce functional elements such as artificial outward-facing promoters that drive expression of genes adjacent to the insertion site or promoter-less genes for the generation of random reporter fusions (8–10). For example, a *mariner*-based transposon, Tn*FLXgfp*, was developed by cloning a promoter-less *gfp* gene, encoding the green fluorescent protein (GFP), and encoding a downstream antibiotic resistance cassette between the two ITRs (11). Thus, a translational fusion between any theoretical target and GFP could be generated provided that the insertion placed the GFP coding sequence in frame. Translational fusions to GFP can be particularly advantageous as the magnitude of fluorescence indicates that gene expression level and subcellular localization patterns, if any, can be informative of gene function. Transposon-delivered reporter systems such as this, however, typically have two primary limitations for retaining function of the target. First, integration of a translational fusion often creates a C-terminal loss-of-function truncation of the target as translation is terminated by the reporter stop codon and downstream antibiotic resistance cassette necessary to select for colonies containing the insertion. Second, the location of the translational fusion is restricted to sites within an open reading frame permitted by the target transposase sequence and the single reading frame dictated by the ITR.

Here, we modify the *in vivo*-delivered Tn*FLX* transposon system in Bacillus subtilis for the generation of internal markerless fusions to fluorescent protein reporters in all three open reading frames. While one of the constructs was nonfunctional due to unavoidable modification of the ITR, the remaining two exhibited transposition at frequencies sufficient for screening using a fluorescence activated cell sorter (FACS). We demonstrate the efficacy of the system by generating internal fluorescent fusions to the essential cell division protein FtsZ expressed in merodiploid at an ectopic site. We further show that the system can generate fusions that are both fluorescent and functional using the flagellar motor protein MotA as a nonessential target. Finally, we modify the transposon to deliver half of a fluorescent protein for random bimolecular fluorescence complementation (BiFC) screening of potential protein interactors with a target bait construct that is translationally coupled to the other half of the fluorophore in the chromosome (12–14). These tools will expand the ability to sample fluorescent fusion integration sites in proteins recalcitrant to reverse genetic fusion design and potentially provide a powerful new approach for unbiased screening of *in vivo* protein-protein interaction studies.

## RESULTS

### Development of transposons to create internal fluorescent protein fusions.

**FIG 1:**
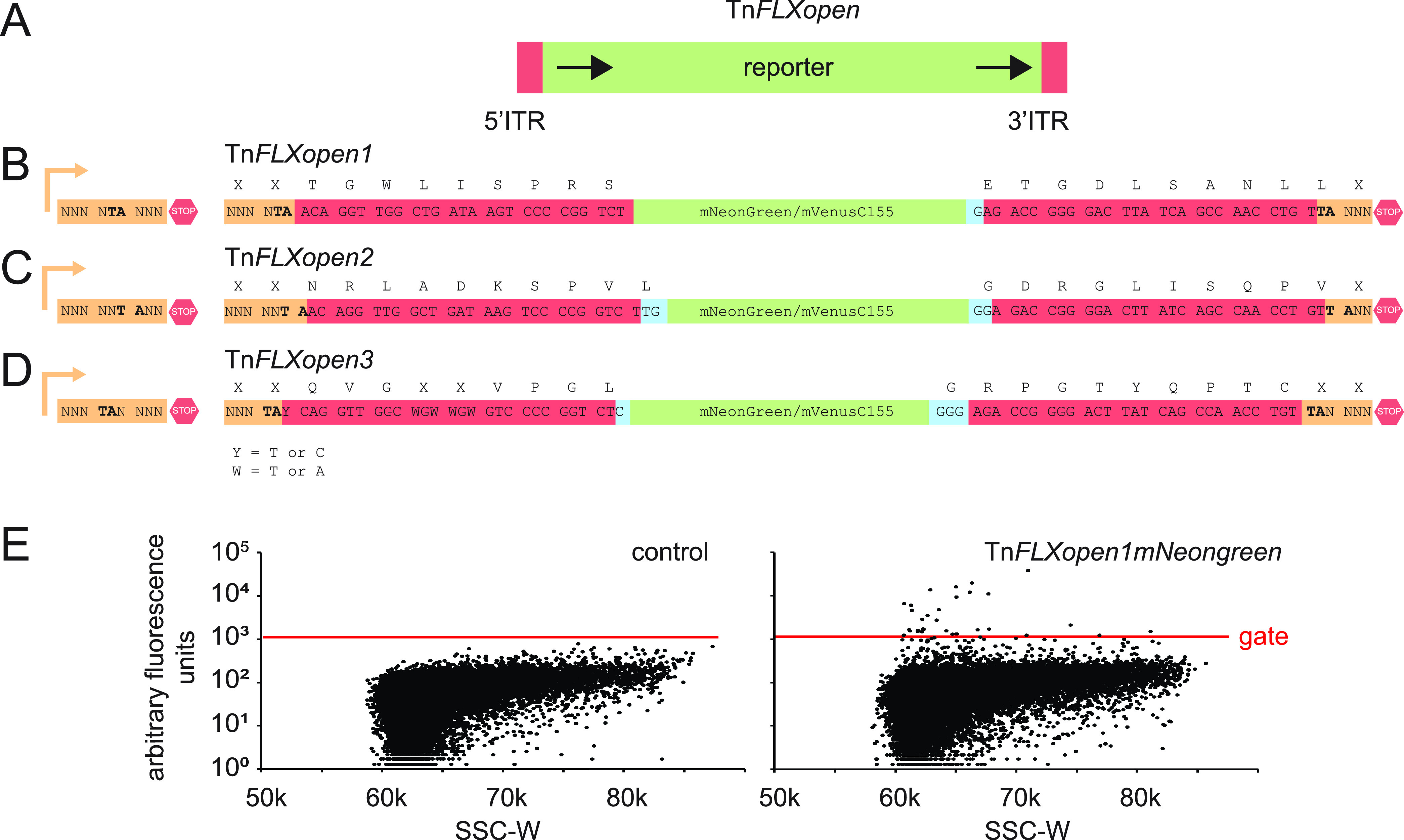
Tn*FLXopen* architecture. (A) The transposons of the Tn*FLXopen* series share an architecture. A reporter construct, either *mNeongreen* or the coding sequence for the C-terminal 85 amino acids of mVenus (colored green), was inserted between two inverted terminal repeat (ITR) sequences for *Himar* transposase recognition (colored red). In each case, the reporter gene lacked a stop codon that would arrest translation within the transposon. (B to D) Left, a cartoon of the theoretical sequence in which the transposon was inserted boxed in orange. Bent arrow indicates a promoter, and stop sign indicates a stop codon. “N” indicates theoretically any nucleotide. Right, a cartoon of the theoretical sequence after the transposon had been inserted in frame. The sequence of the ITR is boxed in red, the sequence of the reporter is boxed in green, and bases added to preserve the open reading frame are boxed in blue. The TA sequence targeted for integration is bolded. Letters above the sequence line indicate the translated amino acid where X is theoretically any residue. (B) The Tn*FLXopen1* transposons were designed to maintain a continuous reading frame when inserted at an AT base doublet that is present at positions 2 and 3 of a codon. (C) The Tn*FLXopen2* transposons were designed to maintain a continuous reading frame when inserted at an AT base doublet that is present at positions 3 and 1, respectively, of two consecutive codons. (D) The Tn*FLXopen3* transposons were designed to maintain a continuous reading frame when inserted at an AT base doublet that is present at positions 1 and 2 of a codon. To avoid premature translation termination, the 5′ ITR was modified at three codon positions with a variety of substitutions. Multiple combinations of ITR substitutions were tested for their ability to permit transposition (Fig. S1). Ultimately, none of the Tn*FLXopen3* series resulted in functional transposition and the series was dropped from further use. (E) FACS sorting results for control (left) and Tn*FLXopen1mNeongreen* mutagenized (right) populations. On the *x* axis is the side scatter width value (SSC-W), and on the *y* axis are the arbitrary fluorescence units. Each panel consists of 30,000 individual events. Red line indicates threshold gate value, and cells emitting fluorescence above this value were selected by FACS.

To overcome the premature truncation of the target protein by the random integration of C-terminal translational fusions, a series of transposons were constructed which, when inserted into an expressed gene, would make in-frame, markerless fusions to the fluorescent protein mNeongreen. The transposons consisted of the *mNeongreen* open reading frame flanked by two inverted terminal repeats (ITRs) recognized by the *Himar* transposase (Tn*FLXopen*) (Fig. 1A). During transposition, the *Himar* transposase relocates the ITRs and intervening DNA into a randomly targeted TA-base doublet elsewhere in the genome (15, 16). If the TA integration site is located within a gene and cooriented with the target, then the transposon insertion could place the upstream ITR and the *mNeongreen* gene in one of three different reading frames. Moreover, during transposition, the TA target site is duplicated such that a TA repeat is found directly adjacent to both the upstream and downstream ITR and the 2-bp insertion would also perturb the open reading frame downstream of the transposon insertion site. Thus, three different transposon constructs were generated to accommodate the three different possible codon positions in which the TA target sequence might fall and preserve the coding frame both upstream and downstream of the transposon insertion site (Fig. 1B to D).

If the TA insertion site is found within the last 2 bp of a codon (e.g., XTA-XXX), the 27-bp *Himar* upstream ITR sequence will permit the open reading frame to proceed unimpeded into the coding sequence of mNeonreen (Fig. 1B). A single base was added to the 3′ end of the *mNeongreen* open reading frame, however, to counterbalance the addition of the TA duplication event and preserve the reading frame (Tn*FLXopen1*) (Fig. 1B). If the TA insertion site straddles two codons (XXT-AXX), the coding region will again extend through the *Himar* upstream ITR unimpeded, but two bases were added to both the 5′ and 3′ ends of the *mNeonreen* gene to preserve the open reading frame (Tn*FLXopen2*) (Fig. 1C). Finally, if the TA insertion site was found at the front of a codon (e.g., XXX-TAX), more modifications were necessary to preserve a continuous open reading frame. Three different mutations of the upstream ITR were necessary to remove stop codons, including an unavoidable change to the first base of the ITR (A), as insertion at a TA doublet would immediately create a UAA stop codon (Fig. S1A). Out of concern that modifications of the ITR motif might diminish transposition frequency, 5 different modifications of the ITR sequence were recombined to create 10 different transposon constructs (Fig. S1B). Finally, one base was added to the 5′ end and three bases were added to the 3′ end of the *mNeongreen* gene to maintain the open reading frame (Tn*FLXopen3*) (Fig. 1D).

To test the Tn*FLXopenmNeongreen* series of transposons, a naturally competent derivative (DK1042) of the undomesticated B. subtilis wild-type strain NCIB 3610 (17) was separately transformed with each of the delivery plasmids. Transposon mutagenesis was conducted by propagating the resulting strains at a temperature permissive for plasmid replication (22°C), selecting for an antibiotic resistance cassette encoded in the plasmid backbone (*erm*), and back diluting the culture for regrowth in media lacking antibiotic at the nonpermissive temperature of 42°C. Note that transposition occurs at a constitutive rate and propagation at the nonpermissive temperature selects against extrachromosomal maintenance of the delivery vehicle to arrest transposon mobility. The resulting transposon libraries were subsequently analyzed by fluorescence microscopy. Cells lacking a transposon delivery system exhibited no fluorescence, but cells mutagenized with either Tn*FLXopen1* or Tn*FLXopen2* contained rare individuals that were fluorescent. Quantitation via flow cytometry allowed us to set a gate threshold based on the nonfluorescent wild type, and approximately 1:5,000 cells in a population mutagenized by Tn*FLXopen1* produced fluorescence above that threshold (Fig. 1E). The 10 variants of Tn*FLXopen3* were incapable of generating fluorescence detectable by microscopy, and flow cytometry indicated that even the best variant produced fluorescent cells at a frequency of perhaps 1:200,000. We infer that the reduced rate of transposition is likely attributed to the substantial genetic modification required to preserve the open reading frame, and due to the low frequency, Tn*FLXopen3* was dropped from further use. Nonetheless, the other two transposons integrated to create fluorescent fusion proteins at a frequency sufficient for screening even in the absence of antibiotic selection.

### Tn*FLXopenmNeongreen* internal fluorescent fusions to FtsZ.

**FIG 2:**
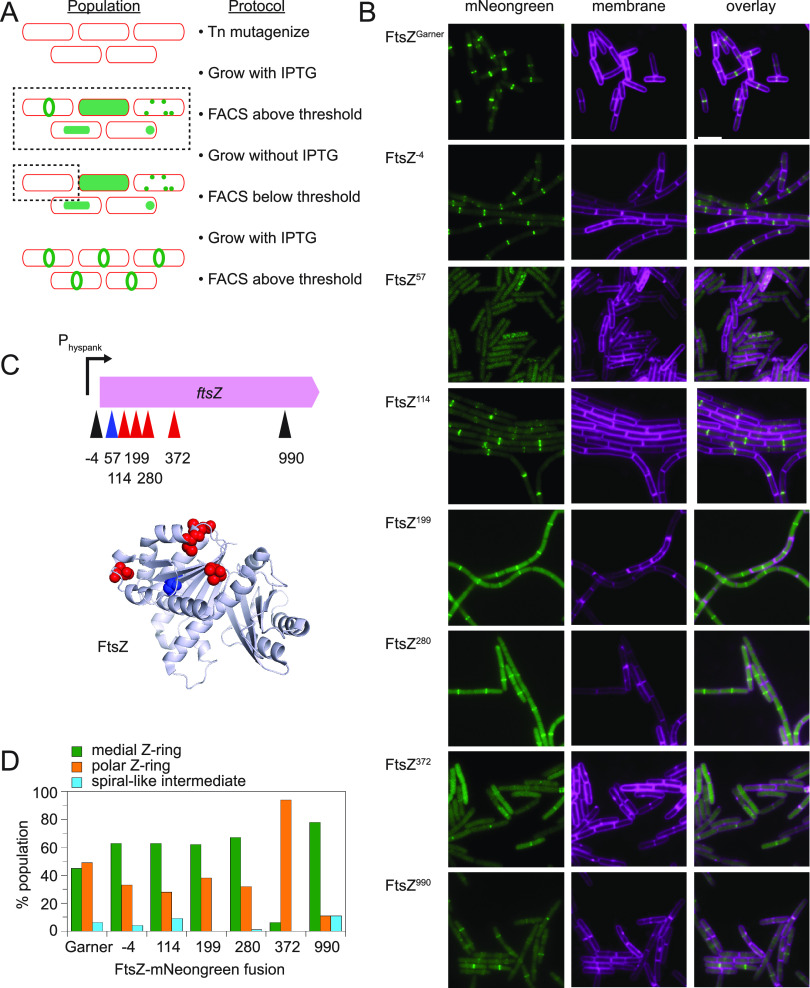
Some Tn*FLXopen* insertions of mNeongreen within FtsZ produce subcellular fluorescence patterns consistent with FtsZ localization. (A) A flow diagram of the selection strategy used to enrich Tn*FLXopenmNeongreen* insertions within *ftsZ*. Cells in a population are indicated by a red outline. After transposition, cells grown in the presence of IPTG were selected for fluorescence by FACS (dotted box), and the population exhibited a diverse variety of fluorescence localization patterns (green). The enriched population was regrown in the absence of IPTG, FACS sorted for nonfluorescent cells (dotted box), and plated. The resulting colonies were regrown in the presence of IPTG, colonies that produced fluorescent cells were retained, and the transposon insertion site within *ftsZ* was determined. (B) Fluorescence microscopy images of the indicated Tn*FLXopenmNeongreen* insertions labeled with the corresponding insertion site. Membranes were stained with FM4-64 (false colored magenta) and FtsZ-mNeongreen (false colored green). The following strains were used to generate this panel: FtsZ^Garner^ (DK5094), FtsZ^−4^ (DK8445), FtsZ^57^ (DK8441), FtsZ^114^ (DK8440), FtsZ^199^ (DK8444), FtsZ^280^ (DK8443), FtsZ^372^ (DK8439), and FtsZ^990^ (DK8442). (C) Top, location of transposon insertion sites within the *ftsZ* gene. Numbers indicate the distance (in bp) from the transposon insertion relative to the translation start site. Red carets indicate insertions predicted to position the mNeongreen fusion on the surface of the protein, blue caret indicates an insertion predicted to position mNeongreen in the FtsZ core region, and black carets indicate insertions that fall within unstructured domains not present in the FtsZ 3-dimensional structure. Bottom, ribbon diagram of the FtsZ protein (PDB 2VAM [60]) from B. subtilis with four of the mNeongreen insertion sites that led to distinct localization patterns space filled in red. Only diffuse localization was observed when insertion of the transposon led to fusion of mNeongreen to the internal core of the FtsZ protein (space filled in blue). AlphaFold2 was used to model each of the resulting fusion proteins (Fig. S2). Each fusion that gave rise to FtsZ-like localization patterns positioned mNeongreen such that it did not interfere with protofilament oligomerization, whereas the one fusion that abolished localization positioned mNeongreen in a steric clash with the adjacent monomer. (D) Localization analysis of cells expressing the various FtsZ-mNeongreen fusion proteins. Green bars represent percentage of events in which the FtsZ ring was observed at the predivisional midcell, orange bars indicate the percentage of events in which the FtsZ ring was observed adjacent to a nascent pole, and cyan bars indicate the percentage of events in which FtsZ adopted a spiral-like intermediate.

The absence of an antibiotic resistance cassette is advantageous as it permits the random generation of fluorescent fusions in the middle of a protein but is disadvantageous for targeted mutagenesis of a protein of interest. To circumvent this problem, a gene of interest whose gene product displays a distinct subcellular localization pattern was expressed from an IPTG (isopropyl-β-d-thiogalactopyranoside)-inducible *P_hyspank_* promoter and inserted at an ectopic locus (*amyE*) with a linked antibiotic resistance cassette. The candidate gene chosen was the *ftsZ* gene encoding the essential cell division protein FtsZ that localizes in a ring-like pattern at the cell division plane (18–21). The resulting strain was grown in the presence of IPTG and mutagenized in parallel with Tn*FLXopen1* and Tn*FLXopen2* to make random insertions, a subset of which generated fusions to FtsZ. To enrich for cells with FtsZ fusions, we first enriched for IPTG-induced cells expressing fluorescent proteins using a fluorescence activated cell sorter (FACS). The majority of cells mutagenized with the transposon exhibited low fluorescence intensity, but rare cells were fluorescent above a threshold and FACS sorting isolated these cells from the pool (Fig. 2A). The sorted population was then repropagated in the absence of IPTG and resorted by FACS, this time selecting for dark cells below the originally selected fluorescence intensity threshold (Fig. 2A). Thus, the probability that a fluorescent fusion was generated in *ftsZ* was increased by sequentially sorting the population for cells with fluorescence that was, like the expression of the *ftsZ* target, IPTG dependent.

To further determine linkage between the fluorescent fusion and the inducible copy of *ftsZ*, an SPP1 phage lysate was generated on the mutant pool and backcrossed by transduction into a wild-type parent selecting for the antibiotic resistance cassette integrated within the ectopic site. Some of the transductant colonies failed to exhibit fluorescence when induced with IPTG and were discarded. We attribute the absence of the enriched phenotype based on the fact that the FACS sorting protocol used was optimized for yield and not stringency and that depending on the nature of each insertion, cells may become nonfluorescent for reasons other than the lack of IPTG induction (e.g., heterogenous gene expression, transient dormancy, etc.). Nonetheless, other transductant colonies exhibited fluorescence upon IPTG induction. Some cells exhibited diffuse cytoplasmic fluorescence after induction, and all but one representative of this class was discarded (Fig. 2B). Sequencing indicated that the location of the transposon 57 bp downstream of the translational start site (FtsZ^57^) would insert the mNeongreen protein sequence near the core of FtsZ structure, potentially disrupting proper folding and producing the diffuse fluorescence observed (Fig. 2C). Fifteen transductants, however, exhibited a subcellular ring-like localization pattern reminiscent of the known pattern of FtsZ, and in each case the inducible copy of *ftsZ* was sequenced to determine the transposon insertion site. Nine of the candidates contained *mNeongreen* insertions that were redundant with others within the class consistent with the propagation of siblings during the enrichment process, and redundant insertions were discarded. Ultimately, six unique insertions in FtsZ were further characterized (Table S1, Fig. 2B). Two of the six were inserted in unresolved and likely disordered regions of the FtsZ protein structure, but the remaining four were inserted at surface-exposed sites perhaps consistent with partial functionality (Fig. 2C).

To determine whether any of the new mNeongreen-FtsZ insertions altered FtsZ localization, each FtsZ localization pattern was compared to a previously established N-terminal mNeongreen fusion to FtsZ, FtsZ^Garner^ (20). In B. subtilis, FtsZ has at least three different localization patterns. First, FtsZ localizes as a medial predivisional ring to recruit late divisome components (22–24). Second, after division is complete, the FtsZ ring dwells at the new polar position until disassembled by the Min system (25–27). Third, a subpopulation of FtsZ may migrate away from the polar ring toward the nascent midcell as a spiral-like intermediate (28, 29). In general, the Tn*FLXopen*-delivered mNeongreen fusions exhibited higher background fluorescence than FtsZ^Garner^, but this may be due to the fact that each of these constructs was expressed by IPTG induction from the artificial *P_hyspank_* promoter in merodiploid whereas FtsZ^Garner^ is expressed in merodiploid from the native *ftsZ* promoter. While the higher background fluorescence in some cases reduced the ability to detect spirals, we note that four of the Tn*FLXopen* fusions (FtsZ^−4^, FtsZ^114^, FtsZ^199^, and FtsZ^280^) exhibited FtsZ localization position frequencies roughly similar to that exhibited by FtsZ^Garner^ (Fig. 2B). One fusion, however, FtsZ^372^, appeared to exhibit preference for polar localization, while another, FtsZ^990^, exhibited preference for medial localization. We conclude that the location of the mNeongreen fusion within FtsZ can alter localization patterns. Moreover, the fusions that favor one ring location over another may inform FtsZ function, either by masking protein interaction sites or by altering the relative rates of protofilament polymerization/depolymerization.

### A Tn*FLXopen* fluorescent and functional fusion to MotA.

**FIG 3:**
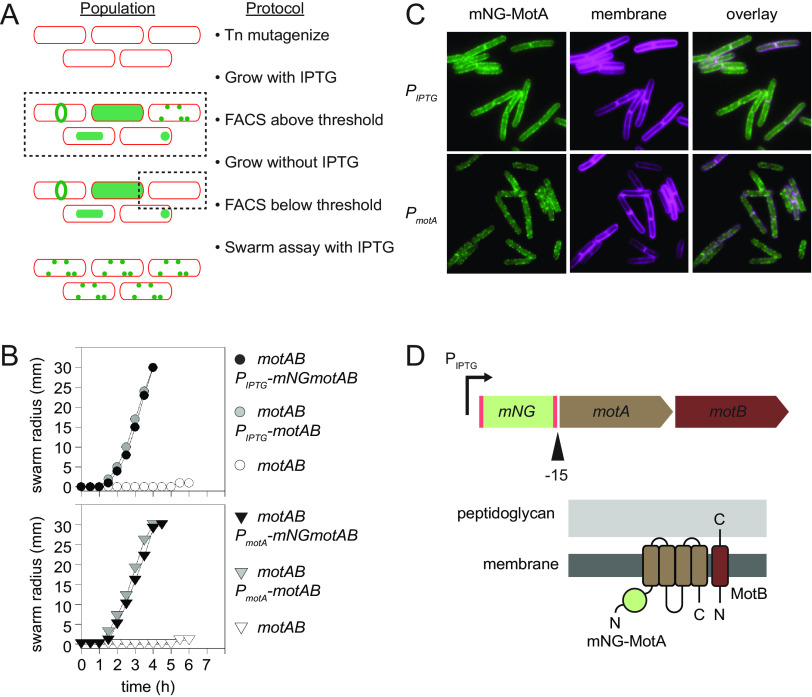
An mNeongreen-MotA fusion complements the lack of the wild-type *motA* gene. (A) A flow diagram of the selection strategy used to enrich Tn*FLXopenmNeongreen* insertions within *motAB*. Cells in a population are indicated by a red outline. After transposition, cells grown in the presence of IPTG were selected for fluorescence by FACS (dotted box) and the population exhibited a diverse variety of fluorescence localization patterns (green). The enriched population was regrown in the absence of IPTG and FACS sorted for nonfluorescent cells (dotted box) and plated. The resulting colonies were regrown in the presence of IPTG, spotted on IPTG-containing swarm agar plates, and cells from motile flares were retained. (B) Quantitative swarm expansion assays. Top, the following strains were measured for motility on swarm agar plates in the presence of IPTG: *motAB* (open circles, DK2530), *motAB* P_IPTG_-*motAB* (gray circles, DK801), *motAB* P_IPTG_*-mNGmotAB* (closed circles, DK6666). Bottom, the following strains were measured for motility on swarm agar plates: *motAB* (open circles, DK2530), *motAB* P*_motA_*-*motAB* (gray circles, DK8678), *motAB* P_motA_-*mNGmotAB* (closed circles, DK7154). *mNG*, *mNeongreen*. Each data point is the average of three replicates. (C) Fluorescence microscopy images of the mNeongreen-MotA (mNG-MotA) fusion expressed from either the IPTG-inducible *P_IPTG_* promoter (top, DK6832) or the native *P_motA_* promoter (bottom, DK7104). Cell membrane was fluorescently stained with FM4-64 (false colored magenta) and mNG-MotA (false colored green). (D) Top, schematic representation of the Tn*FLXopenmNeongreen* transposon inserted 15 bp upstream of the MotA translation start site. Bent arrow represents the IPTG-inducible promoter, red boxes represent inverted terminal repeat regions and the green box represents the *mNeonGreen* gene. Brown boxes represents the *motA* and *motB* genes, respectively, and the black caret indicates the location of the in-frame fusion event generated by transposon insertion. Bottom, model of the mNeongreen-MotA/MotB motor complex in the bacterial membrane. mNeongreen-MotA is represented in light brown with the fused mNeongreen highlighted as a green circle. MotB is colored in dark brown. Lipid bilayer is represented in dark gray, the cell wall in light gray. N and C indicate respective protein termini.

Random fluorescent fusions were generated to FtsZ, but functionality was difficult to assess due to merodiploidy and the essential nature of the target. To screen for random fluorescent fusions that were also functional, a similar strategy was taken with a nonessential target, the flagellar stator proteins MotA and MotB (Fig. 3A) (30–33). A strain doubly mutated for *motA* and *motB* with an IPTG-inducible copy of the *motAmotB* dicistron at an ectopic site (*amyE*) exhibited swarming motility only in the presence of IPTG and was mutagenized with Tn*FLXopenmNeongreen* (Fig. 3B, top). The transposon mutant pool was grown in the presence of IPTG, and FACS was used to select for the fluorescent subpopulation. Next, the subpopulation was regrown in the absence of IPTG and FACS selected for nonfluorescent cells to enrich for individuals in which fluorescence was, like *motAmotB* expression, IPTG dependent (Fig. 3A). Finally, the pool was regrown in the presence of IPTG and spotted in the center of an IPTG-containing swarm agar petri plate to measure motility. Transposants that were nonfunctional for MotA or MotB activity remained nonswarming at the site of inoculation. Cells emerged from the nonmotile population, however, and spread over the surface of the agar. Fifteen clonal isolates were selected by streaking from the swarm edge (Fig. 3B, top).

Microscopic analysis of the candidates grown in the presence of 1 mM IPTG revealed that cells exhibited diffuse fluorescence at the cell membrane with perhaps an indication of puncta within the haze (Fig. 3C). Fluorescent fusions to MotA and MotB in other organisms have exhibited a punctate localization pattern (34–38), and the diffuse staining of the membrane observed here may have been due to the overexpression of the *motAmotB* genes by the inducible promoter. Each candidate was sequenced to determine the location of the transposon insertion site and found to have the exact same insertion in which the *mNeongreen* gene was inserted upstream of, and in frame with, the *motA* gene, thereby generating an N-terminal fusion with a 12-amino acid-long linker sequence (Fig. 3D). Finally, the N-terminal mNeongreen-MotA fusion was shown to complement the swarming motility defect of a *motAmotB* double mutant in the presence of IPTG (39) (Fig. 3B, top). We infer that either the single insertion represents the only functional insertion location within *motAmotB* or multiple functional insertion points are possible and the single recovered insertion was the result of sequential enrichment and propagation of siblings. Whatever the case, one candidate sibling was retained for further study.

To further characterize the mNeongreen-MotA fusion protein, the fusion-containing dicistron was cloned under the control of the native *P_motA_* promoter and inserted at the ectopic *amyE* locus of a *motAmotB* motAB double mutant. Whereas strains deleted for the *motAB* operon have a mucoid colony morphology due to the overexpression of secreted poly-γ-glutamate (30, 31), the mNeongreen-MotAMotB construct produced wild-type-looking colonies perhaps indicative of successful complementation. To quantitatively assess functionality of the *P_motA_-mNeongreen-motAmotB* construct, a swarm expansion assay was performed and the fusion protein was found to be functional as it rescued swarming of the *motAmotB* motAB mutant to wild-type levels (Fig. 3B, bottom). Finally, fluorescence microscopy of liquid-grown cells in exponential phase indicated that the mNeongreen-MotA fusion localized as puncta at the cell membrane consistent with MotA/MotB localization reported for other organisms (Fig. 3C). We conclude that the mNeongreen-MotA fusion is functional and fluorescent. We further conclude that transposon mutagenesis with Tn*FLXopenmNeongreen* is a viable strategy for the unbiased generation and screening of random fusions that are both fluorescent and functional.

### Transposon-mediated markerless fluorescent fusions for detecting protein interactions.

**FIG 4:**
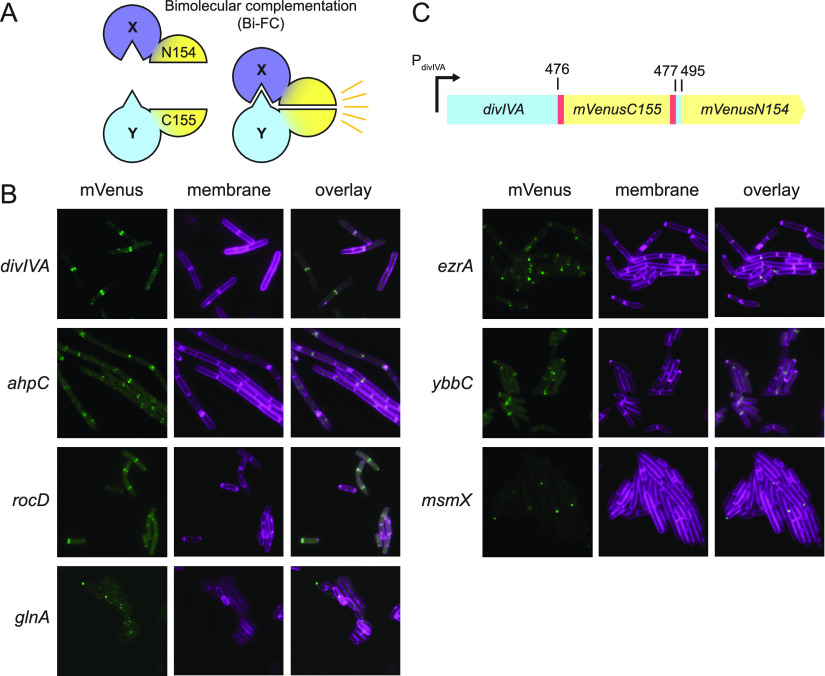
Transposon-delivered BiFC identified candidate DivIVA interactors. (A) Cartoon of bimolecular fluorescence complementation (BiFC) strategy. One half of a fluorophore mVenusN154 is fused to one protein of interest (protein X), and the other half of mVenusC155 is fused to another protein with which protein X interacts (protein Y). The interaction of proteins X and Y bring both mVenus halves into close spatial proximity, thereby restoring fluorophore structure and fluorescence emission. (B) Fluorescence micrographs of Tn*FLXopenmVenusC155* fusion candidates cloned under the control of an IPTG-inducible promoter and inserted at an ectopic locus of a strain expressing DivIVA-mVenusN154 at the native locus. Membrane stained with FM4-64 (false colored in magenta) and mVenus (false colored in green). The following strains were used to generate each panel: *ahpC* (DK8247), *divIVA* (DK8062), *ezrA* (DK8478), *glnA* (DK8179), *msmX* (DK8249), *rocD* (DK8479), and *ybbC* (DK8248). Bar represents 5 μm. (C) Cartoon of the Tn*FLXopenmVenusC155* fusion in the *divIVA-mVenusN154* reporter gene. The transposon inserted the C-terminal half of *mVenus* into the reporter upstream of the N-terminal half of *mVenus*. Blue box indicates *divIVA* sequence, yellow boxes indicate the *mVenus* sequence, and red boxes indicate the Tn*FLXopen Himar* recognition ITR sequences. Numbers above the diagram indicate base pair locations. Bent arrow indicates the *divIVA* promoter.

Most proteins are a part of a cellular network, and their functions are frequently influenced by the proteins with which they interact. Therefore, the determination of proteins in close spatial proximity can help to understand the role of a protein in the cell. One method to detect protein interactions is bimolecular fluorescence complementation (BiFC) in which two halves of a fluorophore are fused to interacting proteins and the protein interaction restores fluorophore structure and function (12–14) (Fig. 4A). Here, we designed an unbiased approach to detect protein interactions using BiFC, random transposon mutagenesis, and high-throughput cell sorting by FACS. Briefly, a protein of interest, in this case the B. subtilis polar scaffolding protein DivIVA (40–43), was fused to the N-terminal 154 amino acids of the fluorophore mVenus (DivIVA-mVenus^1-154^) at the native site in the chromosome. The resulting strain was nonfluorescent due to the mVenus truncation. Next, the strain was mutagenized with derivatives of Tn*FLXopen* in which sequences encoding the 84 C-terminal amino acids of mVenus were integrated between the mariner ITR sequences in the two functional reading frames described previously (Tn*FLXopenmVenus^C155^*). Eight million cells from the transposon mutagenesis were FACS sorted for rare fluorescence, and 100 candidate colonies were recovered. We hypothesized that the transposons generated fusions to one or more proteins that interacted with DivIVA, such that their interaction brought the two domains of mVenus in spatial proximity and generated fluorescence.

Whole-genome sequencing was performed on seven of the fluorescent isolates to determine which transposon, sometimes among multiple, was fused in frame with a target gene candidate (Fig. S3). Each target in-frame fusion was PCR amplified and cloned under the control of an IPTG-inducible promoter to determine whether expression of that fusion alone was sufficient to restore fluorescence in the presence of DivIVA-mVenus^N154^. Seven candidate insertions gave fluorescence upon induction (Fig. 4B). DivIVA dimerizes and forms interdimer contacts to create a polar scaffold-like mesh at the cell pole (44), and consistent with self-interaction, one of the BiFC transposon insertions landed within the DivIVA-mVenus^N154^ construct. The resulting fusion generated a protein in which the C-terminal and N-terminal fragments of mVenus switched order in the primary sequence (Fig. 4C). We infer that DivIVA oligomerization and/or subsequent supermolecular contacts within the mesh restored close proximity of the two fragments of mVenus, sufficient to generate fluorescence. Importantly, the insertion in DivIVA localized to both the nascent septum and the old pole, a pattern consistent with previously reported DivIVA localization (40). Four other transposon insertions in *ezrA* encoding the cell division regulator protein EzrA (45), *rocD* encoding the ornithine transaminase enzyme RocD (46), *ahpC* encoding the alkyl hydroperoxide reductase AhpC (47, 48), and *ybbC* encoding a protein of unknown function also produced fluorescence localization in a manner perhaps consistent with DivIVA (Fig. 4B). Two other insertions in *msmX* encoding the ABC-sugar transporter component MsmX (49) and *glnA* encoding glutamine synthetase GlnA (50) produced fluorescence puncta that were also typically found at the cell pole (Fig. 4B). We conclude that *in vivo*-delivered transposons carrying BiFC complementation fragments are capable of restoring fluorescence, thereby predicting candidate interaction proteins for a protein of interest.

## DISCUSSION

Most translational fusions to fluorescent proteins are made at either the N or C terminus of a target protein for convenience, and such constructs are often sufficient to generate fusions that are both fluorescent and functional. Sometimes, however, N- and/or C-terminal fusions can disrupt or alter protein function, perhaps by interfering with proper folding, occluding protein interaction sites, or concealing regulatory domains like proteolytic degrons. When terminal fusions fail to preserve protein function, the generation of internal in-frame “sandwich” fusions can be advantageous, but the rational design of internal fusions is problematic, particularly when structural information for the protein of interest is absent (26, 51). Here, we modify a *mariner*-based transposon system for the generation of random fluorescent fusion proteins. Briefly, the system abandons the convention of including an antibiotic resistance cassette within the confines of the transposon and allows readthrough of a gene encoding a fluorescent protein when inserted in frame with a target gene in the genome. Instead of selecting for transposants based on growth in the presence of an antibiotic, fluorescence activated cell sorting (FACS) was used to select for fluorescent individuals. With this unbiased approach, the randomness of transposon insertion allows theoretically any location within a protein to be sampled for permissive sites of fusion that are fluorescent and/or functional to overcome the limitations of expectations and convenience (52).

While our transposon-based approach to fluorescent fusion protein generation has advantages, it also has weaknesses, as in the context of the entire genome, random transposon insertion makes recovering insertions in a particular gene of interest rare. Here, we overcome this limitation by a “trap” strategy in which a gene of interest is inserted in the chromosome under the control of an inducible promoter, adjacent to a preexistent antibiotic resistance cassette. Thus, linkage of the fluorescent fusion to the gene of interest can be established by a combination of IPTG-dependent fluorescence and linkage to the preexistent antibiotic resistance. The trap-based approach allowed isolation of a number of insertions in FtsZ that produced localization patterns consistent with FtsZ rings previously established by both fluorescent fusions and immunolocalization. Moreover, a single mNeongreen fusion was recovered for the MotA flagellar stator protein that not only gave a membrane-associated, punctate localization pattern consistent with tagged homologs in other organisms but was also functional for supporting motility when present as the only copy. We conclude that the Tn*FLXopen* system combined with a genetic trap can permit selective retrieval of fluorescent and/or functional fusions to theoretically any protein of interest.

We further expanded the Tn*FLXopen* system for the detection of protein-protein interactions *in vivo* by bimolecular complementation of fluorescence (BiFC). Specifically, half of a fluorophore was fused to a protein of interest and the other half was delivered by transposon insertion. If the insertion generated an in-frame fusion to an interacting partner, the two halves of the fluorophore were brought into functional proximity and that individual clone could be retrieved by FACS. Linkage analysis was problematic due to the absence of an antibiotic resistance cassette linked to the complementary construct, but whole-genome sequencing was sufficient to identify in-frame candidates that could be recloned and tested in isolation. The BiFC system was successful in recovering a known interactor with the polar scaffolding protein DivIVA, namely, DivIVA itself. The approach seemed susceptible to perhaps false-positive interactions with highly abundant metabolic proteins but also yielded other potentially relevant interacting proteins like the cell division regulator EzrA. We conclude that transposon-delivered BiFC was successful at identifying interacting partners with a protein of interest and provides an unbiased *in vivo* approach to protein-protein interaction studies. Moreover, BiFC provides additional protein interaction information at the subcellular level.

Here, we describe a number of applications and strategies for the use of markerless transposons for the generation of random fluorescent protein fusions, but we expect that many other permutations are possible. For example, we imagine that *in vitro* delivery could be used as an alternative to the *in vivo* system, high-throughput microscopic screening of individual colonies could replace FACS analysis, and introduction of a cassette linked to a native gene could serve as a trap for native fluorescent fusion insertion. Whatever the modification, these approaches are not intended to replace rational design, particularly those now aided by protein folding programs, but instead serve as a potentially valuable alternative when such approaches fail, as was the case in generating fusions to the B. subtilis flagellar stator protein complex. Moreover, the tools can be used beyond targeted gene insertion for whole-genome screens such as we demonstrate here by the transposon-delivered BiFC approach for predicting protein interactions. Finally, we anticipate that these tools will be useful in their own right but also spark interest and inspiration in broadening of transposon insertion selections beyond the use of traditional antibiotic resistance cassettes.

## MATERIALS AND METHODS

### Strains and growth conditions.

All strains used in the study are listed in Table 1.

**TABLE 1:** Strains

Strain	Genotype	Tn tag^*a*^
DK1042	*comI^Q12L^* wild type (17)	
PY79	*sfp^0^ swrA^FS^* (61)	
DK801	*motAB::tet amyE::P_hyspank_-motAB spec* (62)	
DK2530	*motAB::tet*	
DK3394	*amyE*::P_hyspank_-*mNeongreen spec*	
DK5094	*mNeongreen-*FtsZ^Garner^	
DK6008	*ΩdivIVA-mVenusN154 cat rocD*::Tn*FLXopenmVenusC155*	TATGCAAGGA
DK6012	*ΩdivIVA-mVenusN154 cat divIVA::*Tn*FLXopenmVenusC155*	TATTTGAGGA
DK6013	*ΩdivIVA-mVenusN154 cat ahpC::*Tn*FLXopenmVenusC155*	TATGCCCAAC
DK6035	*ΩdivIVA-mVenusN154 cat ybbC::TnFLXopenmVenusC155*	TAGAACCTGA
DK6630	*ΩdivIVA-mVenusN154 cat*	
DK6666	*motAB::tet amyE::P_hyspank_-TnFLXopenmNeongreen-motAB spec*	
DK6832	*motAB::tet amyE::P_hyspank_-motAB::TnFLXopenmNeongreen spec*	TAGACAAGCT
DK6833	*motAB::tet amyE::P_hyspank_-motAB::TnFLXopenmNeongreen spec*	TAGACAAGCT
DK7154	*motAB::tet amyE::P_motAB_-mNeongreen-MotAB spec*	
DK7191	*ΩdivIVA-mVenusN154 cat ezrA::TnFLXopenmVenusC155*	TAGATGATGT
DK7196	*ΩdivIVA-mVenusN154 cat msmX::TnFLXopenmVenusC155*	TACATGATGA
DK7197	*ΩdivIVA-mVenusN154 cat glnA::TnFLXopenmVenusC155*	TAGCATGGAG
DK8062	*amyE::P_hyspank_-divIVA-mVenusN154::TnFLXmVenusC155 cat*	TATTTGAGGA
DK8158	*amyE::P_hyspank_ ftsZ kan*	
DK8179	*ΩdivIVA-mVenusN154 cat amyE::_Physpank_ glnA::TnFLXmVenusC155 cat*	TAGCATGGAG
DK8247	*ΩdivIVA-mVenusN154 cat amyE::_Physpank_ ahpC::TnFLXmVenusC155 cat*	TATGCCCAAC
DK8248	*ΩdivIVA-mVenusN154 cat amyE::_Physpank_ ybbC::TnFLXmVenusC155 cat*	TAGAACCTGA
DK8249	*ΩdivIVA-mVenusN154 cat amyE::_Physpank_ msmX::TnFLXmVenusC155 cat*	TACATGATGA
DK8439	*amyE::P_hyspank_ ftsZ^372^TnFLXopenmNeongreen kan*	TAGGCGCATT
DK8440	*amyE::P_hyspank_ ftsZ^114^TnFLXopenmNeongreen kan*	TAGAGTATAT
DK8441	*amyE::P_hyspank_ ftsZ^57^TnFLXopenmNeongreen kan*	TAGGAGGCGG
DK8442	*amyE::P_hyspank_ ftsZ^990^TnFLXopenmNeongreen kan*	TAAATCAAAG
DK8443	*amyE::P_hyspank_ ftsZ^280^TnFLXopenmNeongreen kan*	TAAAGGTGCT
DK8444	*amyE::P_hyspank_ ftsZ^199^TnFLXopenmNeongreen kan*	TAGAGGATTG
DK8445	*amyE::P_hyspank_ ftsZ^−4^TnFLXopenmNeongreen kan*	TAGAATGTTG
DK8478	*ΩdivIVA-mVenusN154 cat amyE::_Physpank_ ezrA::TnFLXmVenusC155 cat*	TAGATGATGT
DK8479	*ΩdivIVA-mVenusN154 cat amyE::_Physpank_ rocD::TnFLXmVenusC155 cat*	TATGCAAGGA
DK8580	*ΩdivIVA-mVenusN154 cat citB::TnFLXopenmVenusC155*	TATTGATGTT
DK8678	*motAB::tet amyE::PmotA-TnFLXopenmNeongreen-motAB spec*	

a Tn tag indicates the nine base pairs adjacent to the transposon insertion site to allow precise determination of transposon position.

B. subtilis and Escherichia coli strains were grown in lysogeny broth (LB; 10 g tryptone, 5 g yeast extract, 5 g NaCl per L) or on LB plates fortified with 1.5% (wt/vol) Bacto agar at 37°C. When appropriate, antibiotics were included at the following concentrations: 10 μg/mL tetracycline, 100 μg/mL spectinomycin, 5 μg/mL chloramphenicol, 5 μg/mL kanamycin, and 1 μg/mL erythromycin plus 25 μg/mL lincomycin (*mls*). Isopropyl-β-d-thiogalactopyranoside (IPTG; Sigma-Aldrich, St. Louis, USA) was added to the medium at the indicated concentration when appropriate.

### Strain creation and maintenance.

All plasmids used in the study are listed in Table S2. All primers used in the study are listed in Table S3. For the construction of the transposon delivery vectors, plasmids were amplified in E. coli DH5α and transferred to E. coli TG1 (*recA^+^*) to generate plasmid concatemers and allow for transformation into B. subtilis.

### Construction of the Tn*FLXopen* delivery plasmids.

To generate Tn*FLXopen1mNeongreen*, the *mNeongreen* gene was equipped with flanking ITRs and a 1-bp spacer distal to the region coding for the fluorophore to maintain the proper reading frame by PCR using chromosomal DNA of DK3394 as a template and oligonucleotides 5680 and 5681. To allow for integration into the universal transposon acceptor plasmid pFK7 (11) at an SmaI restriction site, the obtained fragment was amplified and extended with primers 5759 and 5760 and isothermal assembly (ITA) was performed yielding pFK169 (see Fig. S4 for plasmid sequence).

To generate Tn*FLXopen2mNeongreen*, the *mNeongreen* gene was equipped with flanking ITRs as well as 2-bp spacers proximal and distal to the coding region to maintain the proper reading frame by PCR using chromosomal DNA of DK3394 as a template and oligonucleotides 6949 and 6950. To allow for integration into the universal transposon acceptor plasmid pFK7 at an SmaI restriction site, the obtained fragment was amplified and extended with primers 5759 and 5760 and isothermal assembly was performed creating pFK87 (see Fig. S4 for plasmid sequence).

To generate TnF*LXopen1mVenusC155*, the last 255 bp of the coding region of the *mVenus* gene (*mVenus C155*) was extended with ITRs and a 1-bp spacer distal to the gene fragment to maintain the proper reading frame by PCR amplification using an *mVenus* linear fragment optimized for the codon usage of B. subtilis (synthesized by IDT, Coralville, IA) and oligonucleotides 6146 and 6147. The resulting transposon was re-PCR amplified using primers 5759 and 5760, purified, and integrated into SmaI linearized pFK7 by ITA to create plasmid pFK170 (see Fig. S4 for plasmid sequence).

To generate Tn*FLXopen2mVenusC155*, *mVenusC155* was PCR amplified with oligonucleotides 6953 and 6954, which introduced flanking ITR sequences and two base pair spacers proximal and distal to the gene fragment to maintain reading frame integrity. The resulting transposon was re-PCR amplified using primers 5759 and 5760, purified, and integrated into SmaI linearized pFK7 by ITA to create plasmid pFK110 (see Fig. S4 for plasmid sequence).

### DivIVA-mVenusN154.

A fusion between a truncated *divIVA* gene and the proximal 462 bp of the *mVenus* gene was generated by ITA (53). The *divIVA* fragment was PCR amplified from DK1042 chromosomal DNA using oligonucleotides 6150 and 6151, and the *mVenus* fragment was PCR amplified from a fragment optimized for the codon usage of B. subtilis (synthesized by IDT, Coralville, IA) using oligonucleotides 6152 and 6153 and cloned by ITA into pER19 (54) linearized by SmaI digestion to make pFK86. The pFK86 was isolated from *recA^+^* TG1 E. coli and transformed into DK1042, yielding DK6630.

### P_motA_-mNeongreen-motAmotB.

The *P_motA_-mNeongreen-motAmotB* construct was assembled by amplification of the *P_motA_* sequence with the oligonucleotides 6722 and 6723 using chromosomal DNA of DK1042 as the template and the *mNeongreen-motAmotB* fragment with the oligonucleotides 6724 and 6725 and chromosomal DNA of DK6832. The fragments were restriction-digested with BamHI/SalI and SalI/SphI, respectively, and inserted into BamHI/SphI linearized pAH25 (generous gift of Amy Hitchcock Camp, Mount Holyoke College) by ligation, creating pDP530.

### ITPG-inducible constructs.

The *ftsZ* gene was PCR amplified using DK1042 chromosomal DNA as a template and oligonucleotides 7345 and 7346, purified, and integrated by ITA into pDP111 containing the *P_hyspank_* promoter, a polylinker, and a kanamycin resistance cassette between the arms of the *amyE* genes linearized by SphI to create pFK214 (55). The *ahpC-mVenusC155* gene was PCR amplified using DK6013 chromosomal DNA as a template and oligonucleotides 7365/7366 and integrated by ITA into SalI linearized pDP111 to create pFK222. The *divIVA-mVenusC155-mVenusN154* was PCR amplified using DK6012 chromosomal DNA and with oligonucleotides 7353/7354 and integrated by ITA into SalI linearized pDP111 to create pFK220. The *ezrA-mVenusC155* gene was PCR amplified using DK7191 chromosomal DNA and oligonucleotides 7355/7356 and integrated by ITA into SalI linearized pDP111 to create pFK231. The *glnA-mVenusC155* gene was PCR amplified using DK7197 chromosomal DNA and oligonucleotides 7357/7358 and integrated by ITA into SalI linearized pDP111 to create pFK225. The *msmX-mVenusC155* gene was PCR amplified using DK7196 chromosomal DNA and oligonucleotides 7251/7352 and integrated by ITA into SalI linearized pDP111 to create pFK226. The *rocD-mVenusC155* gene was PCR amplified using DK6008 chromosomal DNA and oligonucleotides 7394/7395 and integrated by ITA into SalI linearized pDP111 to create pFK233. The *ybbC-mVenusC155* gene was PCR amplified using DK6035 chromosomal DNA and oligonucleotides 7361/7362 and integrated by ITA into SalI linearized pDP111 to create pFK223.

### Isothermal assembly reaction.

First, a 5× ITA stock mixture was generated (500 mM Tris-HCl [pH 7.5], 50 mM MgCl_2_, 50 mM dithiothreitol [DTT; Bio-Rad, Hercules, CA, USA], 31.25 mM PEG-8000 [Thermo Fisher Scientific, Waltham, MA, USA], 5.02 mM NAD [Sigma-Aldrich], and 1 mM each deoxynucleoside triphosphate [dNTP; New England BioLabs, Ipswich, USA]), aliquoted, and stored at −80°C. An assembly master mixture was made by combining prepared 5× isothermal assembly reaction buffer (131 mM Tris-HCl, 13.1 mM MgCl_2_, 13.1 mM DTT, 8.21 mM PEG-8000, 1.32 mM NAD, and 0.26 mM each dNTP) with Phusion DNA polymerase (New England BioLabs; 0.033 units/μL), T5 exonuclease diluted 1:5 with 5× reaction buffer (New England BioLabs; 0.01 units/μL), *Taq* DNA ligase (New England BioLabs; 5,328 units/μL), and additional dNTPs (267 μM). The master mix was aliquoted as 15 μL and stored at −80°C. DNA fragments were combined at equimolar amounts to a total volume of 5 μL and added to a 15-μL aliquot of prepared master mix. The reaction mixture was incubated for 60 min at 50°C (53).

### Transposon mutagenesis.

The transposon-harboring plasmids were introduced into the B. subtilis acceptor strain via transformation or SPP1 phage-mediated transduction (56), selecting for *mls* resistance, and incubated at 22°C for 72 h. Subsequently, cells were incubated for 16 h (Tn*FLXopenmNeongreen*) or 30 h (Tn*FLXopenmVenusC155*) at room temperature in LB medium containing *mls*. The longer one incubates cells carrying the delivery plasmid, the more transposition events will occur, and we found that obtaining rare positive BiFC events benefited from longer incubations. After incubation, the cell suspension was diluted 1:100 in LB medium and grown for 6 h at 37°C to cure the cells of the plasmid. Subsequently, the cells were either screened by FACS or inoculated on LB-agar plates.

### Whole-genome sequencing.

Cells were grown in LB medium at 37°C to mid-exponential phase (optical density at 600 nm [OD_600_] of ~0.5) and harvested, and genomic DNA was extracted with the DNeasy blood and tissue kit (Qiagen, Hilden, Germany). Illumina sequencing was performed by the Microbial Genome Sequencing Center (Pittsburgh, PA, USA).

### Transposon insertion site identification by IPCR.

Transposon insertions in the manuscript were identified either by sequencing a known locus or by unbiased whole-genome sequencing. Nonetheless, we developed a system for inverse PCR (IPCR) should one require such a protocol. Cells were grown in LB medium at 37°C for 6 h. DNA was isolated, and 6 μg was digested with 2 U/μg DNA of the restriction endonuclease Sau3AI (New England Biolabs). Ligation with 400 U of T4 DNA ligase (NEB) was performed at room temperature for 2 h with 1.5 μg of the digested DNA. Inverse PCR was performed with 6 μL of ligated DNA using the oligonucleotides 6987 and 6988 for the Tn*FLXopenmNeongreen*, or 6118 and 6119 for the Tn*FLXmVenusC155* system, respectively. The amplified fragment was analyzed by Sanger sequencing (Eurofins Genomics, Louisville, KY, USA) using the oligonucleotide 6987 (Tn*FLXopenmNeongreen*) or 6118 (Tn*FLXmVenusC155*).

### Fluorescence microscopy.

Microscopy was performed with a Nikon eclipse 80i microscope, equipped with a Plan Apo 100× Ph 3 objective (NA 1.4). Cells were mounted on 1% (wt/vol) agarose pads containing S7_50_ minimal medium (57) on object slides. Images were acquired with a Cool snap HQ2 camera (Photometrix) and were processed with Metamorph 7.7.9 software (Universal Imaging Corp.). Membranes were stained with FM4-64 (1 nM final concentration, Molecular Probes).

### Flow cytometry.

Cells were grown in LB medium at 37°C to an OD_600_ of 0.7, sedimented, and resuspended in phosphate-buffered saline (PBS; 137 mM NaCl, 2.7 mM KCl, 10 mM Na_2_HPO_4_, and 2 mM KH_2_PO_4_) at a concentration of 5 × 10^6^ cells/mL. Analysis and cell sorting were performed using a FACSAriaII flow cytometer (BD Biosciences, IUB Flow Cytometry Core Facility) equipped with an 85-μm nozzle. Cells that were separated by FACS were collected in a reaction tube containing LB medium and transferred onto fortified medium for further incubation.

### Swarm expansion assay.

Cells were grown in LB medium to mid-exponential phase at 37°C, sedimented, and resuspended to an OD_600_ of 10 in water supplemented with 0.5% (vol/vol) India ink. Ten microliters of the cell suspension was spotted onto solidified LB agar (0.7% [wt/vol], freshly prepared, and dried for 10 min in a laminar flow hood), dried for 10 min, and incubated at 37°C. Measurements of swarm radius relative to the point of inoculation (demarked by the India ink) were taken every 30 min. IPTG was supplemented to the medium at a concentration of 1 mM when appropriate.

### Protein structure modeling.

Three-dimensional structures were modeled using the ColabFold (58) software, which is based on the structure prediction provided by alphaFold2 (59). The structures were predicted using the following parameters: “Amber” = yes, “templates” = yes, “msa mode” = MMseqs2 (UniRef + Environmental), “model type” = auto, “pair mode” = unpaired + paired, “recycles” = 3.

### Data availability.

All data generated or analyzed during this study are included in this article. Raw data and bacterial strains can be requested from the corresponding authors. Relevant plasmids will be deposited at the Bacillus Genetic Stock Center (BGSC, The Ohio State University, Columbus OH).
