# Supplementary material for: TnFLXopen: Markerless Transposons for Functional Fluorescent Fusion Proteins and Protein Interaction Prediction
Source: Microbiol Spectr. 2022 May 2;10(3):e02428-21. doi: 10.1128/spectrum.02428-21 (PMC9241775; doi:10.1128/spectrum.02428-21)
Supplement: SUPPLEMENTAL FILE 1 — Supplemental material. Download spectrum.02428-21-s001.pdf, PDF file, 0.8 MB [file spectrum.02428-21-s001.pdf]

Table S1: Localization patterns of various mNeongreen insertions in FtsZ

Fusion	Insertion tag	Medial ring	Polar ring	Spiral
$FtsZ^{Garner}$	N/A	78 (45%)	85 (49%)	10 (6%)
$FtsZ^{-4}$	TAGAATGTT	74 (63%)	39 (33%)	5 (4%)
$FtsZ^{57}$	TAGGAGGCG	---	---	---
$FtsZ^{114}$	TAGAGTATAT	67 (63%)	30 (28%)	10 (9%)
$FtsZ^{199}$	TAGAGGATTG	64 (62%)	39 (38%)	0 (0%)
$FtsZ^{280}$	TAAAGGTGCT	78 (67%)	38 (32%)	1 (1%)
$FtsZ^{372}$	TAGGCGCATT	2 (6%)	49 (94%)	0 (0%)
$FtsZ^{990}$	TAAATCAAAG	100 (78%)	14 (11%)	15 (11%)

Table S2: Plasmids

plasmid	relevant genotype	ref
pAH25	*amyE::spec amp*	
pER19	*cat amp*	Ricca 1992
pDP111	*amyE::Physpank kan amp*	Kearns 2005
pDP530	*amyE::*P_{motAB}*-mNeongreen-motAB spec amp*	this study
pDR111	*amyE::*$P_{hyspank}$ *spec amp*	D. Rudner (Harvard U)
pFK7	*mls* $spec^{Ec}$ ori^{BsTs}	Dempwolff 2020
pFK86	*divIVA-mVenusN154 cat amp*	this study
pFK87	Tn*FLXopen2mNeonGreen mls* $spec^{Ec}$ ori^{BsTs}	this study
pFK110	Tn*FLXopen2mVenusC155 mls* $spec^{Ec}$ ori^{BsTs}	this study
pFK169	Tn*FLXopen1mNeonGreen mls* $spec^{Ec}$ ori^{BsTs}	this study
pFK170	Tn*FLXopen1mVenusC155 mls* $spec^{Ec}$ ori^{BsTs}	this study
pFK214	*amyE::*$P_{hyspank}$*-ftsZ, kan amp*	this study
pFK220	*amyE::*$P_{hyspank}$*-divIVAmVenusC155mVenusN154 kan amp*	this study
pFK222	*amyE::*$P_{hyspank}$*-ahpCmVenusC155 kan amp*	this study
pFK223	*amyE::*$P_{hyspank}$*-ybbCmVenusC155 kan amp*	this study
pFK225	*amyE::*$P_{hyspank}$*-glnAmVenusC155 kan amp*	this study
pFK226	*amyE::*$P_{hyspank}$*-msmXmVenusC155 kan amp*	this study
pFK231	*amyE::*$P_{hyspank}$*-ezrAmVenusC155 kan amp*	this study
pFK233	*amyE::*$P_{hyspank}$*-rocDmVenusC155 kan amp*	this study

Table S3: Primers

Primer	Sequence (5’-3’)
5680	ACAGGTTGGCTGATAAGTCCCCGGTCTCCTTATAGAGTTCATCCATACC’
5681	ACAGGTTGGCTGATAAGTCCCCGGTCTATGGTTTCGAAAGGAGAGGAGG
5759	GTAAATAAGTCTAGTGTGCCCACAGGTTGGCTGATAAGTCC
5760	AGCAGCAAGTGTAGGATCCCCACAGGTTGGCTGATAAGTCC
6118	GGATAACGCTCGGTATGGATG
6119	CGCGGTAACAAACTCCAAGAG
6146	ACAGGTTGGCTGATAAGTCCCCGGTCTCCTTGTAA
6147	ACAGGTTGGCTGATAAGTCCCCGGTCTATGGCGGATAAACAAAAAAACGG
6150	TCCGCCACCTTCCTTTTCCTCAAATACAGCG
6151	GTCGACTCTAGAGGATCCCCCCATTAACGCCAAATGATATTC
6152	GAGGAAAAGGAAGGTGGCGGAATGGTCTCTAAAGGAGAAGAATTG
6153	GTGAATTCGAGCTCGGTACCCTTATGTTATGTAGACGTTGTGTGA
6722	AGGAGGGATCCAACCATCCAAGAATGGGTTGAGGA
6723	CTCCTGTCGACAGTTTTCACCAAATCCTTTTTTAC
6724	GATAACAATTAAGCTTAGTCGAC
6725	ATTAGCTTGCATGCGGCTAGC
6949	AGTGTGCCCACAGGTTGGCTGATAAGTCCCCGGTCTTGATGGTTTCGAAAGGAGAGGAGG
6950	TAGGATCCCCACAGGTTGGCTGATAAGTCCCCGGTCTCCCTTATAGAGTTCATCCATACC
6953	GGATCCCCACAGGTTGGCTGATAAGTCCCCGGTCTCCCTTGTAAAGTTCATCCATACCG
6954	GTGCCCACAGGTTGGCTGATAAGTCCCCGGTCTTGATGGCGGATAAACAAAAAAACGGTA
6987	CGGAACTTAATTTTAAAGAGTGGC
6988	CTCGGTTTTTCTGAACACGTAC
7345	GTCGACAGCTAGCCGCATGTAAGGAGGATTTTAGAATGTTGGAGTTCGAAACAAACATAG
7346	CCACCGAATTAGCTTGCATGTTAGCCGCGTTTATTACGGTTTC
7351	GCGGATAACAATTAAGCTTAGCAAAGGGGGATGTTAGATGG
7352	TTAGCTTGCATGCGGCTAGCTGTTATCGGATTCTCACTTCTGTC
7353	TGAGCGGATAACAATTAAGCTTAGGTAAAAATGGAGGTGGCATCATG
7354	TTAGCTTGCATGCGGCTAGCTGTTATGTTATGTAGACGTTGTGTG
7355	GCGGATAACAATTAAGCTTAGGTAAACAGCAAGGGGGGCTC
7356	TTAGCTTGCATGCGGCCTAGCTGCTAAGCGGATATGTCAGCTTTG
7357	GCGGATAACAATTAAGCTTAGAAGAGGAGGAATTTTACCAAATG
7358	TTAGCTTGCATGCGGCTAGCTGTTAATACTGAGACATATACTGTTC
7361	GCGGATAACAATTAAGCTTAGGCTTTAATAAGGAGGGACACATG
7362	TTAGCTTGCATGCGGCTAGCTGCTAATAAATGAGATACTTTTTTCTTG
7365	GCGGATAACAATTAAGCTTAGTTTTAGGAGGAATATACATTATGTC
7366	TTAGCTTGCATGCGGCTAGCTGTTAGATTTTACCTACTAGATCAAG
7395	GAATTAGCTTGCATGCGGCTAGCTGTTATGCGTTTCGCAGCACGTG
7394	CGGATAACAATTAAGCTTAGAGGGAAAGCAGGATAACATGTTG

Table S4 Plasmid sequences

pFK169

247-1039 Spectinomycin resistance confirming cassette (for selection in *E. coli*)

2288-3238 *repA* gene (regulates plasmid copy number in *E. coli*)

3286-3508 origin of replication (for plasmid maintenance in *E. coli*)

3998-5350 temperature sensitive origin of replication (for plasmid maintenance in *B. subtilis*)

5413-6503 Erythromycin resistance confirming cassette (for selection in *B. subtilis*)

6506-7269 Tn*FLXopen1mNeongreen*

7273-8705 σ^B dependent *himar* transposase expression cassette

5’-
AGTAAAGCCCTCGCTAGATTTTAATGCGGATGTTGCGATTACTTCGCCAACTATTGCGATAACAAGAAAAAGCCAG
CCTTTCATGATATATCTCCCAATTTGTGTAGGGCTTATTATGCACGCTTAAAAATAATAAAAGCAGACTTGACCTGA
TAGTTTGGCTGTGAGCAATTATGTGCTTAGTGCATCTAACGCTTGAGTTAAGCCGCGCCGCGAAGCGGCGTCGGCT
TGAACGAATTGTTAGACATTATTTGCCGACTACCTTGGTGATCTCGCCTTTCACGTAGTGGACAAATTCTTCCAACT
GATCTGCGCGCGAGGCCAAGCGATCTTCTTCTTGTCCAAGATAAGCCTGTCTAGCTTCAAGTATGACGGGCTGATA
CTGGGCCGGCAGGCGCTCCATTGCCCAGTCGGCAGCGACATCCTTCGGCGCGATTTTGCCGGTTACTGCGCTGTAC
CAAATGCGGGACAACGTAAGCACTACATTTCGCTCATCGCCAGCCCAGTCGGGCGGCGAGTTCCATAGCGTTAAG
GTTTCATTTAGCGCCTCAAATAGATCCTGTTCAGGAACCGGATCAAAGAGTTCCTCCGCCGCTGGACCTACCAAGG
CAACGCTATGTTCTCTTGCTTTTGTCAGCAAGATAGCCAGATCAATGTCGATCGTGGCTGGCTCGAAGATACCTGC
AAGAATGTCATTGCGCTGCCATTCTCCAAATTGCAGTTCGCGCTTAGCTGGATAACGCCACGGAATGATGTCGTCG
TGCACAACAATGGTGACTTCTACAGCGCGGAGAATCTCGCTCTCTCCAGGGGAAGCCGAAGTTTCCAAAAGGTCG
TTGATCAAAGCTCGCCGCGTTGTTTCATCAAGCCTTACGGTCACCGTAACCAGCAAATCAATATCACTGTGTGGCTT
CAGGCCGCCATCCACTGCGGAGCCGTACAAATGTACGGCCAGCAACGTCGGTTCGAGATGGCGCTCGATGACGCC
AACTACCTCTGATAGTTGAGTCGATACTTCGGCGATCACCGCTTCCCTCATGATGTTTAACTTTGTTTTAGGGCGAC
TGCCCTGCTGCGTAACATCGTTGCTGCTCCATAACATCAAACATCGACCCACGGCGTAACGCGCTTGCTGCTTGGA
TGCCCGAGGCATAGACTGTACCCCAAAAAAACAGTCATAACAAGCCATGAAAACCGCCACTGCGCCGTTACCACC
GCTGCGTTCGGTCAAGGTTCTGGACCAGTTGCGTGAGCGCATACGCTACTTGCATTACAGCTTACGAACCGAACAG
GCTTATGTCCACTGGGTTCGTGCCTTCATCCGTTTCCACGGTGTGCGTCACCCGGCAACCTTGGGCAGCAGCGAAG
TCGAGGCATTTCTGTCCTGGCTGGCGAACGAGCGCAAGGTTTCGGTCTCCACGCATCGTCAGGCATTGGCGGCCTT
GCTGTTCTTCTACGGCAAGGTGCTGTGCACGGATCTGCCCTGGCTTCAGGAGATCGGAAGACCTCGGCCGTCGCG
GCGCTTGCCGGTGGTGCTGACCCCGGATGAAGTGGTTCGCATCCTCGGTTTTCTGGAAGGCGAGCATCGTTTGTTC
GCCCAGCTTCTGTATGGAACGGGCATGCGGATCAGTGAGGGTTTGCAACTGCGGGTCAAGGATCTGGATTTCGAT
CACGGCACGATCATCGTGCGGGAGGGCAAGGGCTCCAAGGATCGGGCCTTGATGTTACCCGAGAGCTTGGCACC
CAGCCTGCGCGAGCAGGGGAATTAATTCCCACGGGTTTTGCTGCCCGCAAACGGGCTGTTCTGGTGTTGCTAGTTT
GTTATCAGAATCGCAGATCCGGCTTCAGGTTTGCCGGCTGAAAGCGCTATTTCTTCCAGAATTGCCATGATTTTTTC
CCCACGGGAGGCGTCACTGGCTCCCGTGTTGTCGGCAGCTTTGATTCGATAAGCAGCATCGCCTGTTTCAGGCTGT
CTATGTGTGACTGTTGAGCTGTAACAAGTTGTCTCAGGTGTTCAATTTCATGTTCTAGTTGCTTTGTTTTACTGGTTT
CACCTGTTCTATTAGGTGTTACATGCTGTTCATCTGTTACATTGTCGATCTGTTCATGGTGAACAGCTTTGAATGCAC
CAAAAACTCGTAAAAGCTCTGATGTATCTATCTTTTTTACACCGTTTTCATCTGTGCATATGGACAGTTTTCCCTTTG

ATATGTAACGGTGAACAGTTGTTCTACTTTTGTTTGTTAGTCTTGATGCTTCACTGATAGATACAAGAGCCATAAGA
ACCTCAGATCCTTCCGTATTTAGCCAGTATGTTCTCTAGTGTGGTTCGTTGTTTTTGCGTGAGCCATGAGAACGAAC
CATTGAGATCATACTTACTTTGCATGTCACTCAAAAATTTTGCCTCAAAACTGGTGAGCTGAATTTTTGCAGTTAAA
GCATCGTGTAGTGTTTTTCTTAGTCCGTTATGTAGGTAGGAATCTGATGTAATGGTTGTTGGTATTTTGTCACCATT
CATTTTTATCTGGTTGTTCTCAAGTTCGGTTACGAGATCCATTTGTCTATCTAGTTCAACTTGGAAAATCAACGTATC
AGTCGGGCGGCCTCGCTTATCAACCACCAATTTCATATTGCTGTAAGTGTTTAAATCTTTACTTATTGGTTTCAAAAC
CCATTGGTTAAGCCTTTTAAACTCATGGTAGTTATTTTCAAGCATTAACATGAACTTAAATTCATCAAGGCTAATCTC
TATATTTGCCTTGTGAGTTTTCTTTTGTGTTAGTTCTTTTAATAACCACTCATAAATCCTCATAGAGTATTTGTTTTCA
AAAGACTTAACATGTTCCAGATTATATTTTATGAATTTTTTTAACTGGAAAAGATAAGGCAATATCTCTTCACTAAA
AACTAATTCTAATTTTTCGCTTGAGAACTTGGCATAGTTTGTCCACTGGAAAATCTCAAAGCCTTTAACCAAAGGAT
TCCTGATTTCCACAGTTCTCGTCATCAGCTCTCTGGTTGCTTTAGCTAATACACCATAAGCATTTTCCCTACTGATGT
TCATCATCTGAGCGTATTGGTTATAAGTGAACGATACCGTCCGTTCTTTCCTTGTAGGGTTTTCAATCGTGGGGTTG
AGTAGTGCCACACAGCATAAAATTAGCTTGGTTTCATGCTCCGTTAAGTCATAGCGACTAATCGCTAGTTCATTTGC
TTTGAAAACAACTAATTCAGACATACATCTCAATTGGTCTAGGTGATTTTAATCACTATACCAATTGAGATGGGCTA
GTCAATGATAATTACTAGTCCTTTTCCTTTGAGTTGTGGGTATCTGTAAATTCTGCTAGACCTTTGCTGGAAAACTT
GTAAATTCTGCTAGACCCTCTGTAAATTCCGCTAGACCTTTGTGTGTTTTTTTTGTTTATATTCAAGTGGTTATAATTT
ATAGAATAAAGAAAGAATAAAAAAAGATAAAAAGAATAGATCCCAGCCCTGTGTATAACTCACTACTTTAGTCAGT
TCCGCAGTATTACAAAAGGATGTCGCAAACGCTGTTTGCTCCTCTACAAAACAGACCTTAAAACCCTAAAGGCTTA
AGTAGCACCCTCGCAAGCTCGGGCAAATCGCTGAATATTCCTTTTGTCTCCGACCATCAGGCACCTGAGTCGCTGT
CTTTTTCGTGACATTCAGTTCGCTGCGCTCACGGCTCTGGCAGTGAATGGGGGTAAATGGCACTACAGGCGCCTTT
TATGGATTCATGCAAGGAAACTACCCATAATACAAGAAAAGCCCGTCACGGGCTTCTCAGGGCGTTTTATGGCGG
GTCTGCTATGTGGTGCTATCTGACTTTTTGCTGTTCAGCAGTTCCTGCCCTCTGATTTTCCAGTCTGACCACTTCGGA
TTATCCCGTGACAGGTCATTCAGACTGGCTAATGCACCCAGTAAGGCAGCGGTATCATCAACAGGCTTACCCGTCT
TACTGTCGGGAATTCCCCGATAGAAAGCGTGAGAAACAGCGTACAGACGATTTAGAGATGTAGAGGTACTTTTAT
GCCGAGAAAACTTTTTGCGTGTGACAGTCCTTAAAATATACTTAGAGCGTAAGCGAAAGTAGTAGCGACAGCTATT
AACTTTCGGTTGCAAAGCTCTAGGATTTTTAATGGACGCAGCGCATCACACGCAAAAAGGAAATTGGAATAAATG
CGAAATTTGAGATGTTAATTAAAGACCTTTTTGAGGTCTTTTTTTCTTAGATTTTTGGGGTTATTTAGGGGAGAAAA
CATAGGGGGGTACTACGACCTCCCCCCTAGGTGTCCATTGTCCATTGTCCAAACAAATAAATAAATATTGGGTTTTT
AATGTTAAAAGGTTGTTTTTTATGTTAAAGTGAAAAAAACAGATGTTGGGAGGTACAGTGATGGTTGTAGATAGA
AAAGAAGAGAAAAAAGTTGCTGTTACTTTAAGACTTACAACAGAAGAAAATGAGATATTAAATAGAATCAAAGAA
AAATATAATATTAGCAAATCAGATGCAACCGGTATTCTAATAAAAAAATATGCAAAGGAGGAATACGGTGCATTTT
AAACAAAAAAAGATAGACAGCACTGGCATGCTGCCTATCTATGACTAAATTTTGTTAAGTGTATTAGCACCGTTATT
ATATCATGAGCGAAAATGTAATAAAAGAAACTGAAAACAAGAAAAATTCAAGAGGACGTAATTGGACATTTGTTT
TATATCCAGAATCAGCAAAAGCCGAGTGGTTAGAGTATTTAAAAGAGTTACACATTCAATTTGTAGTGTCTCCATT
ACATGATAGGGATACTGATACAGAAGGTAGGATGAAAAAAGAGCATTATCATATTCTAGTGATGTATGAGGGTAA
TAAATCTTATGAACAGATAAAAATAATTAACAGAAGAATTGAATGCGACTATTCCGCAGATTGCAGGAAGTGTGA
AAGGTCTTGTGAGATATATGCTTCACATGGACGATCCTAATAAATTTAAATATCAAAAAGAAGATATGATAGTTTA
TGGCGGTGTAGATGTTGATGAATTATTAAAGAAAACAACAACAGATAGATATAAATTAATTAAAGAAATGATTGA
GTTTATTGATGAACAAGGAATCGTAGAATTTAAGAGTTTAATGGATTATGCAATGAAGTTTAAATTTGATGATTGG
TTCCCGCTTTTATGTGATAACTCGGCGTATGTTATTCAAGAATATATAAAATCAAATCGGTATAAATCTGACCGATA
GATTTTGAATTTAGGTGTCACAAGACACTCTTTTTTCGCACCAGCGAAAACTGGTTTAAGCCGACTGCGCAAAAGA
CATAATCGATTCACAAAAAATAGGCACACGAAAAACAAGTTAAGGGATGCAGTTTATGCATCCCTTAACTTACTTA
TTAAATAATTTATAGCTATTGAAAAGAGATAAGAATTGTTCAAAGCTAATATTGTTTAAATCGTCAATTCCTGCATG
TTTTAAGGAATTGTTAAATTGATTTTTTGTAAATATTTTCTTGTATTCTTTGTTAACCCATTTCATAACGAAATAATTA

TACTTTTGTTTATCTTTGTGTGATATTCTTGATTTTTTTCTACTTAATCTGATAAGTGAGCTATTCACTTTAGGTTTAG
GATGAAAATATTCTCTTGGAACCATACTTAATATAGAAATATCAACTTCTGCCATTAAAAGTAATGCCAATGAGCGT
TTTGTATTTAATAATCTTTTAGCAAACCCGTATTCCACGATTAAATAAATCTCATTAGCTATACTATCAAAAACAATT
TTGCGTATTATATCCGTACTTATGTTATAAGGTATATTACCATATATTTTATAGGATTGGTTTTTAGGAAATTTAAAC
TGCAATATATCCTTGTTTAAAACTTGGAAATTATCGTGATCAACAAGTTTATTTTCTGTAGTTTTGCATAATTTATGG
TCTATTTCAATGGCAGTTACGAAATTACACCTCTTTACTAATTCAAGGGTAAAATGGCCTTTTCCTGAGCCGATTTCA
AAGATATTATCATGTTCATTTAATCTTATATTTGTCATTATTTTATCTATATTATGTTTTGAAGTAATAAAGTTTTGAC
TGTGTTTTATATTTTTCTCGTTCATTATAACCCTCTTTAATTTGGTTATATGAATTTTGCTTATTAACGATTCATTATAA
CCACTTATTTTTTGTTTGGTTGATAATGAACTGTGCTGATTACAAAAATACTAAAAATGCCCATATTTTTTCCTCCTTA
TAAAATTAGTATAATTATAGCACGAGCTCTGATAAATATGAACATGATGAGTGATCGTTAAATTTATACTGCAATCG
GATGCGATTATTGAATAAAAGATATGAGAGATTTATCTAATTTCTTTTTTCTTGTAAAAAAAGAAAGTTCTTAAAGG
TTTTATAGTTTTGGTCGTAGAGCACACGGTTTAACGACTTAATTACGAAGTAAATAAGTCTAGTGTGCCCACAGGTT
GGCTGATAAGTCCCCGGTCTATGGTTTCGAAAGGAGAGGAGGATAATATGGCTAGCCTCCCAGCGACCCACGAAC
TGCATATTTTTGGCAGCATTAATGGCGTTGACTTTGATATGGTGGGGCAGGGAACAGGGAACCCTAACGATGGCT
ATGAGGAGCTCAATCTCAAGAGTACAAAAGGAGATTTGCAATTTTCACCTTGGATCCTGGTTCCGCATATTGGCTA
CGGCTTTCATCAATACTTGCCTTATCCGGACGGCATGTCCCCGTTCCAAGCTGCGATGGTGGATGGTTCTGGGTAC
CAGGTGCACCGTACTATGCAGTTTGAGGACGGTGCCTCACTGACGGTCAACTATAGATATACTTATGAAGGCTCAC
ACATTAAGGGTGAGGCCCAAGTTAAAGGAACAGGGTTTCCTGCGGATGGACCGGTAATGACAAACAGTTTAACCG
CTGCGGACTGGTGTCGCTCGAAAAAAACATACCCAAACGATAAAACGATCATCTCGACCTTCAAATGGAGCTATAC
TACGGGCAACGGCAAACGCTATCGTTCCACAGCACGCACGACTTATACGTTTGCTAAACCGATGGCCGCAAACTAC
CTCAAAAATCAACCTATGTACGTGTTCAGAAAAACCGAGTTAAAACATTCAAAAACGGAACTTAATTTTAAAGAGT
GGCAAAAGGCGTTTACAGACGTGATGGGTATGGATGAACTCTATAAGGAGACCGGGGACTTATCAGCCAACCTGT
GGGGATCCTACACTTGCTGCTAATGCGCTCGTTGAAAACGGAGCGAAAGAAGTATATGCATGCTGTACACACCCT
GTACTATCAGGCCCTGCGGTTGAACGGATTAATAATTCAACAATTAAAGAGCTTGTTGTGACAAACAGCATCAAGC
TTCCTGAAGAAAAGAAAATTGAACGCTTTAAGCAGCTTTCAGTCGGACCGCTTCTGGCCGAAGCGATTATTCGCGT
TCATGAGCAGCAATCAGTCAGCTATCTGTTCAGCTAAACCATTTTTCGAGGTTTAAATCCTTATCGTTATGGGTATT
GTTTGTAATAGGACAACTAAAACGACAAGAGGTCGACTAAGGAGGTGAGAGCAATGGAAAAAAAGGAATTTCGT
GTTTTGATAAAATACTGTTTTCTGAAGGGAAAAAATACAGTGGAAGCAAAAACTTGGCTTGATAATGAGTTTCCGG
ACTCTGCCCCAGGGAAATCAACAATAATTGATTGGTATGCAAAATTCAAGCGTGGTGAAATGAGCACGGAGGACG
GTGAACGCAGTGGACGCCCGAAAGAGGTGGTTACCGACGAAAACATCAAAAAAATCCACAAAATGATTTTGAATG
ACCGTAAAATGAAGTTGATCGAGATAGCAGAGGCCTTAAAGATATCAAAGGAACGTGTTGGTCATATCATTCATC
AATATTTGGATATGCGGAAGCTCTGTGCGAAATGGGTGCCGCGCGAGCTCACATTTGACCAAAAACAACGACGTG
TTGATGATTCTAAGCGGTGTTTGCAGCTGTTAACTCGTAATACACCCGAGTTTTTCCGTCGATATGTGACAATGGAT
GAAACATGGCTCCATCACTACACTCCTGAGTCCAATCGACAGTCGGCTGAGTGGACAGCGACCGGTGAACCGTCT
CCGAAGCGTGGAAAGACTCAAAAGTCCGCTGGCAAAGTAATGGCCTCTGTTTTTTGGGATGCGCATGGAATAATT
TTTATCGATTATCTTGAGAAGGGAAAAACCATCAACAGTGACTATTATATGGCGTTATTGGAGCGTTTGAAGGTCG
AAATCGCGGCAAAACGGCCCCACATGAAGAAGAAAAAAGTGTTGTTCCACCAAGACAACGCACCGTGCCACAAGT
CATTGAGAACGATGGCAAAAATTCATGAATTGGGCTTCGAATTGCTTCCCCACCCGCCGTATTCTCCAGATCTGGC
CCCCAGCGACTTTTTCTTGTTCTCAGACCTCAAAAGGATGCTCGCAGGGAAAAAATTTGGCTGCAATGAAGAGGTG
ATCGCCGAAACTGAGGCCTATTTTGAGGCAAAACCGAAGGAGTACTACCAAAATGGTATCAAAAAATTGGAAGGT
CGTTATAATCGTTGTATCGCTCTTGAAGGGAACTATGTTGAATAATAAAGGGGATCCGTCGACCTGCAGCCAAGCT
T-3’

pFK87

247-1039 Spectinomycin resistance confirming cassette (for selection in *E. coli*)

2288-3238 *repA* gene (regulates plasmid copy number in *E. coli*)

3286-3508 origin of replication (for plasmid maintenance in *E. coli*)

3998-5350 temperature sensitive origin of replication (for plasmid maintenance in *B. subtilis*)

5413-6503 Erythromycin resistance confirming cassette (for selection in *B. subtilis*)

6506-7272 Tn*FLXopen2mNeongreen*

7276-8708 σ^B dependent *himar* transposase expression cassette

5'-
AGTAAAGCCCTCGCTAGATTTTAATGCGGATGTTGCGATTACTTCGCCAACTATTGCGATAACAAGAAAAAGCCAG
CCTTTCATGATATATCTCCCAATTTGTGTAGGGCTTATTATGCACGCTTAAAAATAATAAAAGCAGACTTGACCTGA
TAGTTTGGCTGTGAGCAATTATGTGCTTAGTGCATCTAACGCTTGAGTTAAGCCGCGCCGCGAAGCGGCGTCGGCT
TGAACGAATTGTTAGACATTATTTGCCGACTACCTTGGTGATCTCGCCTTTCACGTAGTGGACAAATTCTTCCAACT
GATCTGCGCGCGAGGCCAAGCGATCTTCTTCTTGTCCAAGATAAGCCTGTCTAGCTTCAAGTATGACGGGCTGATA
CTGGGCCGGCAGGCGCTCCATTGCCCAGTCGGCAGCGACATCCTTCGGCGCGATTTTGCCGGTTACTGCGCTGTAC
CAAATGCGGGACAACGTAAGCACTACATTTCGCTCATCGCCAGCCCAGTCGGGCGGCGAGTTCCATAGCGTTAAG
GTTTCATTTAGCGCCTCAAATAGATCCTGTTCAGGAACCGGATCAAAGAGTTCCTCCGCCGCTGGACCTACCAAGG
CAACGCTATGTTCTCTTGCTTTTGTCAGCAAGATAGCCAGATCAATGTCGATCGTGGCTGGCTCGAAGATACCTGC
AAGAATGTCATTGCGCTGCCATTCTCCAAATTGCAGTTCGCGCTTAGCTGGATAACGCCACGGAATGATGTCGTCG
TGCACAACAATGGTGACTTCTACAGCGCGGAGAATCTCGCTCTCTCCAGGGGAAGCCGAAGTTTCCAAAAGGTCG
TTGATCAAAGCTCGCCGCGTTGTTTCATCAAGCCTTACGGTCACCGTAACCAGCAAATCAATATCACTGTGTGGCTT
CAGGCCGCCATCCACTGCGGAGCCGTACAAATGTACGGCCAGCAACGTCGGTTCGAGATGGCGCTCGATGACGCC
AACTACCTCTGATAGTTGAGTCGATACTTCGGCGATCACCGCTTCCCTCATGATGTTTAACTTTGTTTTAGGGCGAC
TGCCCTGCTGCGTAACATCGTTGCTGCTCCATAACATCAAACATCGACCCACGGCGTAACGCGCTTGCTGCTTGGA
TGCCCGAGGCATAGACTGTACCCCAAAAAAACAGTCATAACAAGCCATGAAAACCGCCACTGCGCCGTTACCACC
GCTGCGTTCGGTCAAGGTTCTGGACCAGTTGCGTGAGCGCATACGCTACTTGCATTACAGCTTACGAACCGAACAG
GCTTATGTCCACTGGGTTCGTGCCTTCATCCGTTTCCACGGTGTGCGTCACCCGGCAACCTTGGGCAGCAGCGAAG
TCGAGGCATTTCTGTCCTGGCTGGCGAACGAGCGCAAGGTTTCGGTCTCCACGCATCGTCAGGCATTGGCGGCCTT
GCTGTTCTTCTACGGCAAGGTGCTGTGCACGGATCTGCCCTGGCTTCAGGAGATCGGAAGACCTCGGCCGTCGCG
GCGCTTGCCGGTGGTGCTGACCCCGGATGAAGTGGTTCGCATCCTCGGTTTTCTGGAAGGCGAGCATCGTTTGTTC
GCCCAGCTTCTGTATGGAACGGGCATGCGGATCAGTGAGGGTTTGCAACTGCGGGTCAAGGATCTGGATTTCGAT
CACGGCACGATCATCGTGCGGGAGGGCAAGGGCTCCAAGGATCGGGCCTTGATGTTACCCGAGAGCTTGGCACC
CAGCCTGCGCGAGCAGGGGAATTAATTCCCACGGGTTTTGCTGCCCGCAAACGGGCTGTTCTGGTGTTGCTAGTTT
GTTATCAGAATCGCAGATCCGGCTTCAGGTTTGCCGGCTGAAAGCGCTATTTCTTCCAGAATTGCCATGATTTTTTC
CCCACGGGAGGCGTCACTGGCTCCCGTGTTGTCGGCAGCTTTGATTCGATAAGCAGCATCGCCTGTTTCAGGCTGT
CTATGTGTGACTGTTGAGCTGTAACAAGTTGTCTCAGGTGTTCAATTTCATGTTCTAGTTGCTTTGTTTTACTGGTTT
CACCTGTTCTATTAGGTGTTACATGCTGTTCATCTGTTACATTGTCGATCTGTTCATGGTGAACAGCTTTGAATGCAC
CAAAAACTCGTAAAAGCTCTGATGTATCTATCTTTTTTACACCGTTTTCATCTGTGCATATGGACAGTTTTCCCTTTG
ATATGTAACGGTGAACAGTTGTTCTACTTTTGTTTGTTAGTCTTGATGCTTCACTGATAGATACAAGAGCCATAAGA
ACCTCAGATCCTTCCGTATTTAGCCAGTATGTTCTCTAGTGTGGTTCGTTGTTTTTGCGTGAGCCATGAGAACGAAC

CATTGAGATCATACTTACTTTGCATGTCACTCAAAAATTTTGCCTCAAAACTGGTGAGCTGAATTTTTGCAGTTAAA
GCATCGTGTAGTGTTTTTCTTAGTCCGTTATGTAGGTAGGAATCTGATGTAATGGTTGTTGGTATTTTGTCACCATT
CATTTTTATCTGGTTGTTCTCAAGTTCGGTTACGAGATCCATTTGTCTATCTAGTTCAACTTGGAAAATCAACGTATC
AGTCGGGCGGCCTCGCTTATCAACCACCAATTTCATATTGCTGTAAGTGTTTAAATCTTTACTTATTGGTTTCAAAAC
CCATTGGTTAAGCCTTTTAAACTCATGGTAGTTATTTTCAAGCATTAACATGAACTTAAATTCATCAAGGCTAATCTC
TATATTTGCCTTGTGAGTTTTCTTTTGTGTTAGTTCTTTTAATAACCACTCATAAATCCTCATAGAGTATTTGTTTTCA
AAAGACTTAACATGTTCCAGATTATATTTTATGAATTTTTTTAACTGGAAAAGATAAGGCAATATCTCTTCACTAAA
AACTAATTCTAATTTTTCGCTTGAGAACTTGGCATAGTTTGTCCACTGGAAAATCTCAAAGCCTTTAACCAAAGGAT
TCCTGATTTCCACAGTTCTCGTCATCAGCTCTCTGGTTGCTTTAGCTAATACACCATAAGCATTTTCCCTACTGATGT
TCATCATCTGAGCGTATTGGTTATAAGTGAACGATACCGTCCGTTCTTTCCTTGTAGGGTTTTCAATCGTGGGGTTG
AGTAGTGCCACACAGCATAAAATTAGCTTGGTTTCATGCTCCGTTAAGTCATAGCGACTAATCGCTAGTTCATTTGC
TTTGAAAACAACTAATTCAGACATACATCTCAATTGGTCTAGGTGATTTTAATCACTATACCAATTGAGATGGGCTA
GTCAATGATAATTACTAGTCCTTTTCCTTTGAGTTGTGGGTATCTGTAAATTCTGCTAGACCTTTGCTGGAAAACTT
GTAAATTCTGCTAGACCCTCTGTAAATTCCGCTAGACCTTTGTGTGTTTTTTTTGTTTATATTCAAGTGGTTATAATTT
ATAGAATAAAGAAAGAATAAAAAAAGATAAAAAGAATAGATCCCAGCCCTGTGTATAACTCACTACTTTAGTCAGT
TCCGCAGTATTACAAAAGGATGTCGCAAACGCTGTTTGCTCCTCTACAAAACAGACCTTAAAACCCTAAAGGCTTA
AGTAGCACCCTCGCAAGCTCGGGCAAATCGCTGAATATTCCTTTTGTCTCCGACCATCAGGCACCTGAGTCGCTGT
CTTTTTCGTGACATTCAGTTCGCTGCGCTCACGGCTCTGGCAGTGAATGGGGGTAAATGGCACTACAGGCGCCTTT
TATGGATTCATGCAAGGAAACTACCCATAATACAAGAAAAGCCCGTCACGGGCTTCTCAGGGCGTTTTATGGCGG
GTCTGCTATGTGGTGCTATCTGACTTTTTGCTGTTCAGCAGTTCCTGCCCTCTGATTTTCCAGTCTGACCACTTCGGA
TTATCCCGTGACAGGTCATTCAGACTGGCTAATGCACCCAGTAAGGCAGCGGTATCATCAACAGGCTTACCCGTCT
TACTGTCGGGAATTCCCCGATAGAAAGCGTGAGAAACAGCGTACAGACGATTTAGAGATGTAGAGGTACTTTTAT
GCCGAGAAAACTTTTTGCGTGTGACAGTCCTTAAAATATACTTAGAGCGTAAGCGAAAGTAGTAGCGACAGCTATT
AACTTTCGGTTGCAAAGCTCTAGGATTTTTAATGGACGCAGCGCATCACACGCAAAAAGGAAATTGGAATAAATG
CGAAATTTGAGATGTTAATTAAAGACCTTTTTGAGGTCTTTTTTTCTTAGATTTTTGGGGTTATTTAGGGGAGAAAA
CATAGGGGGGTACTACGACCTCCCCCCTAGGTGTCCATTGTCCATTGTCCAAACAAATAAATAAATATTGGGTTTTT
AATGTTAAAAGGTTGTTTTTTATGTTAAAGTGAAAAAAACAGATGTTGGGAGGTACAGTGATGGTTGTAGATAGA
AAAGAAGAGAAAAAAGTTGCTGTTACTTTAAGACTTACAACAGAAGAAAATGAGATATTAAATAGAATCAAAGAA
AAATATAATATTAGCAAATCAGATGCAACCGGTATTCTAATAAAAAAATATGCAAAGGAGGAATACGGTGCATTTT
AAACAAAAAAAGATAGACAGCACTGGCATGCTGCCTATCTATGACTAAATTTTGTTAAGTGTATTAGCACCGTTATT
ATATCATGAGCGAAAATGTAATAAAAGAAACTGAAAACAAGAAAAATTCAAGAGGACGTAATTGGACATTTGTTT
TATATCCAGAATCAGCAAAAGCCGAGTGGTTAGAGTATTTAAAAGAGTTACACATTCAATTTGTAGTGTCTCCATT
ACATGATAGGGATACTGATACAGAAGGTAGGATGAAAAAAGAGCATTATCATATTCTAGTGATGTATGAGGGTAA
TAAATCTTATGAACAGATAAAAATAATTAACAGAAGAATTGAATGCGACTATTCCGCAGATTGCAGGAAGTGTGA
AAGGTCTTGTGAGATATATGCTTCACATGGACGATCCTAATAAATTTAAATATCAAAAAGAAGATATGATAGTTTA
TGGCGGTGTAGATGTTGATGAATTATTAAAGAAAACAACAACAGATAGATATAAATTAATTAAAGAAATGATTGA
GTTTATTGATGAACAAGGAATCGTAGAATTTAAGAGTTTAATGGATTATGCAATGAAGTTTAAATTTGATGATTGG
TTCCCGCTTTTATGTGATAACTCGGCGTATGTTATTCAAGAATATATAAAATCAAATCGGTATAAATCTGACCGATA
GATTTTGAATTTAGGTGTCACAAGACACTCTTTTTTCGCACCAGCGAAAACTGGTTTAAGCCGACTGCGCAAAAGA
CATAATCGATTCACAAAAAATAGGCACACGAAAAACAAGTTAAGGGATGCAGTTTATGCATCCCTTAACTTACTTA
TTAAATAATTTATAGCTATTGAAAAGAGATAAGAATTGTTCAAAGCTAATATTGTTTAAATCGTCAATTCCTGCATG
TTTTAAGGAATTGTTAAATTGATTTTTTGTAAATATTTTCTTGTATTCTTTGTTAACCCATTTCATAACGAAATAATTA
TACTTTTGTTTATCTTTGTGTGATATTCTTGATTTTTTTCTACTTAATCTGATAAGTGAGCTATTCACTTTAGGTTTAG
GATGAAAATATTCTCTTGGAACCATACTTAATATAGAAATATCAACTTCTGCCATTAAAAGTAATGCCAATGAGCGT

TTTGTATTTAATAATCTTTTAGCAAACCCGTATTCCACGATTAAATAAATCTCATTAGCTATACTATCAAAAACAATT
TTGCGTATTATATCCGTACTTATGTTATAAGGTATATTACCATATATTTTATAGGATTGGTTTTTAGGAAATTTAAAC
TGCAATATATCCTTGTTTAAAACTTGGAAATTATCGTGATCAACAAGTTTATTTTCTGTAGTTTTGCATAATTTATGG
TCTATTTCAATGGCAGTTACGAAATTACACCTCTTTACTAATTCAAGGGTAAAATGGCCTTTTCCTGAGCCGATTTCA
AAGATATTATCATGTTCATTTAATCTTATATTTGTCATTATTTTATCTATATTATGTTTTGAAGTAATAAAGTTTTGAC
TGTGTTTTATATTTTTCTCGTTCATTATAACCCTCTTTAATTTGGTTATATGAATTTTGCTTATTAACGATTCATTATAA
CCACTTATTTTTTGTTTGGTTGATAATGAACTGTGCTGATTACAAAAATACTAAAAATGCCCATATTTTTTCCTCCTTA
TAAAATTAGTATAATTATAGCACGAGCTCTGATAAATATGAACATGATGAGTGATCGTTAAATTTATACTGCAATCG
GATGCGATTATTGAATAAAAGATATGAGAGATTTATCTAATTTCTTTTTTCTTGTAAAAAAAGAAAGTTCTTAAAGG
TTTTATAGTTTTGGTCGTAGAGCACACGGTTTAACGACTTAATTACGAAGTAAATAAGTCTAGTGTGCCCACAGGTT
GGCTGATAAGTCCCCGGTCTTGATGGTTTCGAAAGGAGAGGAGGATAATATGGCTAGCCTCCCAGCGACCCACGA
ACTGCATATTTTTGGCAGCATTAATGGCGTTGACTTTGATATGGTGGGGCAGGGAACAGGGAACCCTAACGATGG
CTATGAGGAGCTCAATCTCAAGAGTACAAAAGGAGATTTGCAATTTTCACCTTGGATCCTGGTTCCGCATATTGGC
TACGGCTTTCATCAATACTTGCCTTATCCGGACGGCATGTCCCCGTTCCAAGCTGCGATGGTGGATGGTTCTGGGT
ACCAGGTGCACCGTACTATGCAGTTTGAGGACGGTGCCTCACTGACGGTCAACTATAGATATACTTATGAAGGCTC
ACACATTAAGGGTGAGGCCCAAGTTAAAGGAACAGGGTTTCCTGCGGATGGACCGGTAATGACAAACAGTTTAAC
CGCTGCGGACTGGTGTCGCTCGAAAAAAACATACCCAAACGATAAAACGATCATCTCGACCTTCAAATGGAGCTAT
ACTACGGGCAACGGCAAACGCTATCGTTCCACAGCACGCACGACTTATACGTTTGCTAAACCGATGGCCGCAAACT
ACCTCAAAAATCAACCTATGTACGTGTTCAGAAAAACCGAGTTAAAACATTCAAAAACGGAACTTAATTTTAAAGA
GTGGCAAAAGGCGTTTACAGACGTGATGGGTATGGATGAACTCTATAAGGGAGACCGGGGACTTATCAGCCAAC
CTGTGGGGATCCTACACTTGCTGCTAATGCGCTCGTTGAAAACGGAGCGAAAGAAGTATATGCATGCTGTACACA
CCCTGTACTATCAGGCCCTGCGGTTGAACGGATTAATAATTCAACAATTAAAGAGCTTGTTGTGACAAACAGCATC
AAGCTTCCTGAAGAAAAGAAAATTGAACGCTTTAAGCAGCTTTCAGTCGGACCGCTTCTGGCCGAAGCGATTATTC
GCGTTCATGAGCAGCAATCAGTCAGCTATCTGTTCAGCTAAACCATTTTTCGAGGTTTAAATCCTTATCGTTATGGG
TATTGTTTGTAATAGGACAACTAAAACGACAAGAGGTCGACTAAGGAGGTGAGAGCAATGGAAAAAAAGGAATT
TCGTGTTTTGATAAAATACTGTTTTCTGAAGGGAAAAAATACAGTGGAAGCAAAAACTTGGCTTGATAATGAGTTT
CCGGACTCTGCCCCAGGGAAATCAACAATAATTGATTGGTATGCAAAATTCAAGCGTGGTGAAATGAGCACGGAG
GACGGTGAACGCAGTGGACGCCCGAAAGAGGTGGTTACCGACGAAAACATCAAAAAAATCCACAAAATGATTTT
GAATGACCGTAAAATGAAGTTGATCGAGATAGCAGAGGCCTTAAAGATATCAAAGGAACGTGTTGGTCATATCAT
TCATCAATATTTGGATATGCGGAAGCTCTGTGCGAAATGGGTGCCGCGCGAGCTCACATTTGACCAAAAACAACG
ACGTGTTGATGATTCTAAGCGGTGTTTGCAGCTGTTAACTCGTAATACACCCGAGTTTTTCCGTCGATATGTGACAA
TGGATGAAACATGGCTCCATCACTACACTCCTGAGTCCAATCGACAGTCGGCTGAGTGGACAGCGACCGGTGAAC
CGTCTCCGAAGCGTGGAAAGACTCAAAAGTCCGCTGGCAAAGTAATGGCCTCTGTTTTTTGGGATGCGCATGGAA
TAATTTTTATCGATTATCTTGAGAAGGGAAAAACCATCAACAGTGACTATTATATGGCGTTATTGGAGCGTTTGAA
GGTCGAAATCGCGGCAAAACGGCCCCACATGAAGAAGAAAAAAGTGTTGTTCCACCAAGACAACGCACCGTGCC
ACAAGTCATTGAGAACGATGGCAAAAATTCATGAATTGGGCTTCGAATTGCTTCCCCACCCGCCGTATTCTCCAGA
TCTGGCCCCCAGCGACTTTTTCTTGTTCTCAGACCTCAAAAGGATGCTCGCAGGGAAAAAATTTGGCTGCAATGAA
GAGGTGATCGCCGAAACTGAGGCCTATTTTGAGGCAAAACCGAAGGAGTACTACCAAAATGGTATCAAAAAATTG
GAAGGTCGTTATAATCGTTGTATCGCTCTTGAAGGGAACTATGTTGAATAATAAAGGGGATCCGTCGACCTGCAG
CCAAGCTT-3’

pFK170

247-1039 Spectinomycin resistance confirming cassette (for selection in *E. coli*)

2288-3238 *repA* gene (regulates plasmid copy number in *E. coli*)

3286-3508 origin of replication (for plasmid maintenance in *E. coli*)

3998-5350 temperature sensitive origin of replication (for plasmid maintenance in *B. subtilis*)

5413-6503 Erythromycin resistance confirming cassette (for selection in *B. subtilis*)

6506-6819 Tn*FLXopen1mVenusC155*

6822-8255 σ^B dependent *himar* transposase expression cassette

5’-
AGTAAAGCCCTCGCTAGATTTTAATGCGGATGTTGCGATTACTTCGCCAACTATTGCGATAACAAGAAAAAGCCAG
CCTTTCATGATATATCTCCCAATTTGTGTAGGGCTTATTATGCACGCTTAAAAATAATAAAAGCAGACTTGACCTGA
TAGTTTGGCTGTGAGCAATTATGTGCTTAGTGCATCTAACGCTTGAGTTAAGCCGCGCCGCGAAGCGGCGTCGGCT
TGAACGAATTGTTAGACATTATTTGCCGACTACCTTGGTGATCTCGCCTTTCACGTAGTGGACAAATTCTTCCAACT
GATCTGCGCGCGAGGCCAAGCGATCTTCTTCTTGTCCAAGATAAGCCTGTCTAGCTTCAAGTATGACGGGCTGATA
CTGGGCCGGCAGGCGCTCCATTGCCCAGTCGGCAGCGACATCCTTCGGCGCGATTTTGCCGGTTACTGCGCTGTAC
CAAATGCGGGACAACGTAAGCACTACATTTCGCTCATCGCCAGCCCAGTCGGGCGGCGAGTTCCATAGCGTTAAG
GTTTCATTTAGCGCCTCAAATAGATCCTGTTCAGGAACCGGATCAAAGAGTTCCTCCGCCGCTGGACCTACCAAGG
CAACGCTATGTTCTCTTGCTTTTGTCAGCAAGATAGCCAGATCAATGTCGATCGTGGCTGGCTCGAAGATACCTGC
AAGAATGTCATTGCGCTGCCATTCTCCAAATTGCAGTTCGCGCTTAGCTGGATAACGCCACGGAATGATGTCGTCG
TGCACAACAATGGTGACTTCTACAGCGCGGAGAATCTCGCTCTCTCCAGGGGAAGCCGAAGTTTCCAAAAGGTCG
TTGATCAAAGCTCGCCGCGTTGTTTCATCAAGCCTTACGGTCACCGTAACCAGCAAATCAATATCACTGTGTGGCTT
CAGGCCGCCATCCACTGCGGAGCCGTACAAATGTACGGCCAGCAACGTCGGTTCGAGATGGCGCTCGATGACGCC
AACTACCTCTGATAGTTGAGTCGATACTTCGGCGATCACCGCTTCCCTCATGATGTTTAACTTTGTTTTAGGGCGAC
TGCCCTGCTGCGTAACATCGTTGCTGCTCCATAACATCAAACATCGACCCACGGCGTAACGCGCTTGCTGCTTGGA
TGCCCGAGGCATAGACTGTACCCCAAAAAAACAGTCATAACAAGCCATGAAAACCGCCACTGCGCCGTTACCACC
GCTGCGTTCGGTCAAGGTTCTGGACCAGTTGCGTGAGCGCATACGCTACTTGCATTACAGCTTACGAACCGAACAG
GCTTATGTCCACTGGGTTCGTGCCTTCATCCGTTTCCACGGTGTGCGTCACCCGGCAACCTTGGGCAGCAGCGAAG
TCGAGGCATTTCTGTCCTGGCTGGCGAACGAGCGCAAGGTTTCGGTCTCCACGCATCGTCAGGCATTGGCGGCCTT
GCTGTTCTTCTACGGCAAGGTGCTGTGCACGGATCTGCCCTGGCTTCAGGAGATCGGAAGACCTCGGCCGTCGCG
GCGCTTGCCGGTGGTGCTGACCCCGGATGAAGTGGTTCGCATCCTCGGTTTTCTGGAAGGCGAGCATCGTTTGTTC
GCCCAGCTTCTGTATGGAACGGGCATGCGGATCAGTGAGGGTTTGCAACTGCGGGTCAAGGATCTGGATTTCGAT
CACGGCACGATCATCGTGCGGGAGGGCAAGGGCTCCAAGGATCGGGCCTTGATGTTACCCGAGAGCTTGGCACC
CAGCCTGCGCGAGCAGGGGAATTAATTCCCACGGGTTTTGCTGCCCGCAAACGGGCTGTTCTGGTGTTGCTAGTTT
GTTATCAGAATCGCAGATCCGGCTTCAGGTTTGCCGGCTGAAAGCGCTATTTCTTCCAGAATTGCCATGATTTTTTC
CCCACGGGAGGCGTCACTGGCTCCCGTGTTGTCGGCAGCTTTGATTCGATAAGCAGCATCGCCTGTTTCAGGCTGT
CTATGTGTGACTGTTGAGCTGTAACAAGTTGTCTCAGGTGTTCAATTTCATGTTCTAGTTGCTTTGTTTTACTGGTTT
CACCTGTTCTATTAGGTGTTACATGCTGTTCATCTGTTACATTGTCGATCTGTTCATGGTGAACAGCTTTGAATGCAC
CAAAAACTCGTAAAAGCTCTGATGTATCTATCTTTTTTACACCGTTTTCATCTGTGCATATGGACAGTTTTCCCTTTG

ATATGTAACGGTGAACAGTTGTTCTACTTTTGTTTGTTAGTCTTGATGCTTCACTGATAGATACAAGAGCCATAAGA
ACCTCAGATCCTTCCGTATTTAGCCAGTATGTTCTCTAGTGTGGTTCGTTGTTTTTGCGTGAGCCATGAGAACGAAC
CATTGAGATCATACTTACTTTGCATGTCACTCAAAAATTTTGCCTCAAAACTGGTGAGCTGAATTTTTGCAGTTAAA
GCATCGTGTAGTGTTTTTCTTAGTCCGTTATGTAGGTAGGAATCTGATGTAATGGTTGTTGGTATTTTGTCACCATT
CATTTTTATCTGGTTGTTCTCAAGTTCGGTTACGAGATCCATTTGTCTATCTAGTTCAACTTGGAAAATCAACGTATC
AGTCGGGCGGCCTCGCTTATCAACCACCAATTTCATATTGCTGTAAGTGTTTAAATCTTTACTTATTGGTTTCAAAAC
CCATTGGTTAAGCCTTTTAAACTCATGGTAGTTATTTTCAAGCATTAACATGAACTTAAATTCATCAAGGCTAATCTC
TATATTTGCCTTGTGAGTTTTCTTTTGTGTTAGTTCTTTTAATAACCACTCATAAATCCTCATAGAGTATTTGTTTTCA
AAAGACTTAACATGTTCCAGATTATATTTTATGAATTTTTTTAACTGGAAAAGATAAGGCAATATCTCTTCACTAAA
AACTAATTCTAATTTTTCGCTTGAGAACTTGGCATAGTTTGTCCACTGGAAAATCTCAAAGCCTTTAACCAAAGGAT
TCCTGATTTCCACAGTTCTCGTCATCAGCTCTCTGGTTGCTTTAGCTAATACACCATAAGCATTTTCCCTACTGATGT
TCATCATCTGAGCGTATTGGTTATAAGTGAACGATACCGTCCGTTCTTTCCTTGTAGGGTTTTCAATCGTGGGGTTG
AGTAGTGCCACACAGCATAAAATTAGCTTGGTTTCATGCTCCGTTAAGTCATAGCGACTAATCGCTAGTTCATTTGC
TTTGAAAACAACTAATTCAGACATACATCTCAATTGGTCTAGGTGATTTTAATCACTATACCAATTGAGATGGGCTA
GTCAATGATAATTACTAGTCCTTTTCCTTTGAGTTGTGGGTATCTGTAAATTCTGCTAGACCTTTGCTGGAAAACTT
GTAAATTCTGCTAGACCCTCTGTAAATTCCGCTAGACCTTTGTGTGTTTTTTTTGTTTATATTCAAGTGGTTATAATTT
ATAGAATAAAGAAAGAATAAAAAAAGATAAAAAGAATAGATCCCAGCCCTGTGTATAACTCACTACTTTAGTCAGT
TCCGCAGTATTACAAAAGGATGTCGCAAACGCTGTTTGCTCCTCTACAAAACAGACCTTAAAACCCTAAAGGCTTA
AGTAGCACCCTCGCAAGCTCGGGCAAATCGCTGAATATTCCTTTTGTCTCCGACCATCAGGCACCTGAGTCGCTGT
CTTTTTCGTGACATTCAGTTCGCTGCGCTCACGGCTCTGGCAGTGAATGGGGGTAAATGGCACTACAGGCGCCTTT
TATGGATTCATGCAAGGAAACTACCCATAATACAAGAAAAGCCCGTCACGGGCTTCTCAGGGCGTTTTATGGCGG
GTCTGCTATGTGGTGCTATCTGACTTTTTGCTGTTCAGCAGTTCCTGCCCTCTGATTTTCCAGTCTGACCACTTCGGA
TTATCCCGTGACAGGTCATTCAGACTGGCTAATGCACCCAGTAAGGCAGCGGTATCATCAACAGGCTTACCCGTCT
TACTGTCGGGAATTCCCCGATAGAAAGCGTGAGAAACAGCGTACAGACGATTTAGAGATGTAGAGGTACTTTTAT
GCCGAGAAAACTTTTTGCGTGTGACAGTCCTTAAAATATACTTAGAGCGTAAGCGAAAGTAGTAGCGACAGCTATT
AACTTTCGGTTGCAAAGCTCTAGGATTTTTAATGGACGCAGCGCATCACACGCAAAAAGGAAATTGGAATAAATG
CGAAATTTGAGATGTTAATTAAAGACCTTTTTGAGGTCTTTTTTTCTTAGATTTTTGGGGTTATTTAGGGGAGAAAA
CATAGGGGGGTACTACGACCTCCCCCCTAGGTGTCCATTGTCCATTGTCCAAACAAATAAATAAATATTGGGTTTTT
AATGTTAAAAGGTTGTTTTTTATGTTAAAGTGAAAAAAACAGATGTTGGGAGGTACAGTGATGGTTGTAGATAGA
AAAGAAGAGAAAAAAGTTGCTGTTACTTTAAGACTTACAACAGAAGAAAATGAGATATTAAATAGAATCAAAGAA
AAATATAATATTAGCAAATCAGATGCAACCGGTATTCTAATAAAAAAATATGCAAAGGAGGAATACGGTGCATTTT
AAACAAAAAAAGATAGACAGCACTGGCATGCTGCCTATCTATGACTAAATTTTGTTAAGTGTATTAGCACCGTTATT
ATATCATGAGCGAAAATGTAATAAAAGAAACTGAAAACAAGAAAAATTCAAGAGGACGTAATTGGACATTTGTTT
TATATCCAGAATCAGCAAAAGCCGAGTGGTTAGAGTATTTAAAAGAGTTACACATTCAATTTGTAGTGTCTCCATT
ACATGATAGGGATACTGATACAGAAGGTAGGATGAAAAAAGAGCATTATCATATTCTAGTGATGTATGAGGGTAA
TAAATCTTATGAACAGATAAAAATAATTAACAGAAGAATTGAATGCGACTATTCCGCAGATTGCAGGAAGTGTGA
AAGGTCTTGTGAGATATATGCTTCACATGGACGATCCTAATAAATTTAAATATCAAAAAGAAGATATGATAGTTTA
TGGCGGTGTAGATGTTGATGAATTATTAAAGAAAACAACAACAGATAGATATAAATTAATTAAAGAAATGATTGA
GTTTATTGATGAACAAGGAATCGTAGAATTTAAGAGTTTAATGGATTATGCAATGAAGTTTAAATTTGATGATTGG
TTCCCGCTTTTATGTGATAACTCGGCGTATGTTATTCAAGAATATATAAAATCAAATCGGTATAAATCTGACCGATA
GATTTTGAATTTAGGTGTCACAAGACACTCTTTTTTCGCACCAGCGAAAACTGGTTTAAGCCGACTGCGCAAAAGA
CATAATCGATTCACAAAAAATAGGCACACGAAAAACAAGTTAAGGGATGCAGTTTATGCATCCCTTAACTTACTTA
TTAAATAATTTATAGCTATTGAAAAGAGATAAGAATTGTTCAAAGCTAATATTGTTTAAATCGTCAATTCCTGCATG
TTTTAAGGAATTGTTAAATTGATTTTTTGTAAATATTTTCTTGTATTCTTTGTTAACCCATTTCATAACGAAATAATTA

TACTTTTGTTTATCTTTGTGTGATATTCTTGATTTTTTTCTACTTAATCTGATAAGTGAGCTATTCACTTTAGGTTTAG
GATGAAAATATTCTCTTGGAACCATACTTAATATAGAAATATCAACTTCTGCCATTAAAAGTAATGCCAATGAGCGT
TTTGTATTTAATAATCTTTTAGCAAACCCGTATTCCACGATTAAATAAATCTCATTAGCTATACTATCAAAAACAATT
TTGCGTATTATATCCGTACTTATGTTATAAGGTATATTACCATATATTTTATAGGATTGGTTTTTAGGAAATTTAAAC
TGCAATATATCCTTGTTTAAAACTTGGAAATTATCGTGATCAACAAGTTTATTTTCTGTAGTTTTGCATAATTTATGG
TCTATTTCAATGGCAGTTACGAAATTACACCTCTTTACTAATTCAAGGGTAAAATGGCCTTTTCCTGAGCCGATTTCA
AAGATATTATCATGTTCATTTAATCTTATATTTGTCATTATTTTATCTATATTATGTTTTGAAGTAATAAAGTTTTGAC
TGTGTTTTATATTTTTCTCGTTCATTATAACCCTCTTTAATTTGGTTATATGAATTTTGCTTATTAACGATTCATTATAA
CCACTTATTTTTTGTTTGGTTGATAATGAACTGTGCTGATTACAAAAATACTAAAAATGCCCATATTTTTTCCTCCTTA
TAAAATTAGTATAATTATAGCACGAGCTCTGATAAATATGAACATGATGAGTGATCGTTAAATTTATACTGCAATCG
GATGCGATTATTGAATAAAAGATATGAGAGATTTATCTAATTTCTTTTTTCTTGTAAAAAAAGAAAGTTCTTAAAGG
TTTTATAGTTTTGGTCGTAGAGCACACGGTTTAACGACTTAATTACGAAGTAAATAAGTCTAGTGTGCCCACAGGTT
GGCTGATAAGTCCCCGGTCTatgGCGGATAAACAAAAAAACGGTATTAAAGCAAACTTCAAGATCCGGCATAATAT
TGAAGATGGGGGTGTCCAGCTCGCCGACCACTACCAACAAAACACTCCGATAGGTGACGGGCCAGTGTTACTGCC
GGACAATCATTACCTTAGCTACCAAAGCAAGTTGAGCAAGGACCCTAATGAGAAACGGGATCACATGGTACTCTT
GGAGTTTGTTACCGCGGCTGGGATAACGCTCGGTATGGATGAACTTTACAAGGAGACCGGGGACTTATCAGCCAA
CCTGTGGGGATCCTACACTTGCTGCTAATGCGCTCGTTGAAAACGGAGCGAAAGAAGTATATGCATGCTGTACAC
ACCCTGTACTATCAGGCCCTGCGGTTGAACGGATTAATAATTCAACAATTAAAGAGCTTGTTGTGACAAACAGCAT
CAAGCTTCCTGAAGAAAAGAAAATTGAACGCTTTAAGCAGCTTTCAGTCGGACCGCTTCTGGCCGAAGCGATTATT
CGCGTTCATGAGCAGCAATCAGTCAGCTATCTGTTCAGCTAAACCATTTTTCGAGGTTTAAATCCTTATCGTTATGG
GTATTGTTTGTAATAGGACAACTAAAACGACAAGAGGTCGACTAAGGAGGTGAGAGCAATGGAAAAAAAGGAAT
TTCGTGTTTTGATAAAATACTGTTTTCTGAAGGGAAAAAATACAGTGGAAGCAAAAACTTGGCTTGATAATGAGTT
TCCGGACTCTGCCCCAGGGAAATCAACAATAATTGATTGGTATGCAAAATTCAAGCGTGGTGAAATGAGCACGGA
GGACGGTGAACGCAGTGGACGCCCGAAAGAGGTGGTTACCGACGAAAACATCAAAAAAATCCACAAAATGATTT
TGAATGACCGTAAAATGAAGTTGATCGAGATAGCAGAGGCCTTAAAGATATCAAAGGAACGTGTTGGTCATATCA
TTCATCAATATTTGGATATGCGGAAGCTCTGTGCGAAATGGGTGCCGCGCGAGCTCACATTTGACCAAAAACAAC
GACGTGTTGATGATTCTAAGCGGTGTTTGCAGCTGTTAACTCGTAATACACCCGAGTTTTTCCGTCGATATGTGACA
ATGGATGAAACATGGCTCCATCACTACACTCCTGAGTCCAATCGACAGTCGGCTGAGTGGACAGCGACCGGTGAA
CCGTCTCCGAAGCGTGGAAAGACTCAAAAGTCCGCTGGCAAAGTAATGGCCTCTGTTTTTTGGGATGCGCATGGA
ATAATTTTTATCGATTATCTTGAGAAGGGAAAAACCATCAACAGTGACTATTATATGGCGTTATTGGAGCGTTTGA
AGGTCGAAATCGCGGCAAAACGGCCCCACATGAAGAAGAAAAAAGTGTTGTTCCACCAAGACAACGCACCGTGC
CACAAGTCATTGAGAACGATGGCAAAAATTCATGAATTGGGCTTCGAATTGCTTCCCCACCCGCCGTATTCTCCAG
ATCTGGCCCCCAGCGACTTTTTCTTGTTCTCAGACCTCAAAAGGATGCTCGCAGGGAAAAAATTTGGCTGCAATGA
AGAGGTGATCGCCGAAACTGAGGCCTATTTTGAGGCAAAACCGAAGGAGTACTACCAAAATGGTATCAAAAAATT
GGAAGGTCGTTATAATCGTTGTATCGCTCTTGAAGGGAACTATGTTGAATAATAAAGGGGATCCGTCGACCTGCA
GCCAAGCTT-3’

pFK110

247-1039 Spectinomycin resistance confirming cassette (for selection in *E. coli*)

2288-3238 *repA* gene (regulates plasmid copy number in *E. coli*)

3286-3508 origin of replication (for plasmid maintenance in *E. coli*)

3998-5350 temperature sensitive origin of replication (for plasmid maintenance in *B. subtilis*)

5413-6503 Erythromycin resistance confirming cassette (for selection in *B. subtilis*)

6506-6822 Tn*FLXopen2mVenusC155*

6827-8258 σ^B dependent *himar* transposase expression cassette

5'-
AGTAAAGCCCTCGCTAGATTTTAATGCGGATGTTGCGATTACTTCGCCAACTATTGCGATAACAAGAAAAAGCCAG
CCTTTCATGATATATCTCCCAATTTGTGTAGGGCTTATTATGCACGCTTAAAAATAATAAAAGCAGACTTGACCTGA
TAGTTTGGCTGTGAGCAATTATGTGCTTAGTGCATCTAACGCTTGAGTTAAGCCGCGCCGCGAAGCGGCGTCGGCT
TGAACGAATTGTTAGACATTATTTGCCGACTACCTTGGTGATCTCGCCTTTCACGTAGTGGACAAATTCTTCCAACT
GATCTGCGCGCGAGGCCAAGCGATCTTCTTCTTGTCCAAGATAAGCCTGTCTAGCTTCAAGTATGACGGGCTGATA
CTGGGCCGGCAGGCGCTCCATTGCCCAGTCGGCAGCGACATCCTTCGGCGCGATTTTGCCGGTTACTGCGCTGTAC
CAAATGCGGGACAACGTAAGCACTACATTTCGCTCATCGCCAGCCCAGTCGGGCGGCGAGTTCCATAGCGTTAAG
GTTTCATTTAGCGCCTCAAATAGATCCTGTTCAGGAACCGGATCAAAGAGTTCCTCCGCCGCTGGACCTACCAAGG
CAACGCTATGTTCTCTTGCTTTTGTCAGCAAGATAGCCAGATCAATGTCGATCGTGGCTGGCTCGAAGATACCTGC
AAGAATGTCATTGCGCTGCCATTCTCCAAATTGCAGTTCGCGCTTAGCTGGATAACGCCACGGAATGATGTCGTCG
TGCACAACAATGGTGACTTCTACAGCGCGGAGAATCTCGCTCTCTCCAGGGGAAGCCGAAGTTTCCAAAAGGTCG
TTGATCAAAGCTCGCCGCGTTGTTTCATCAAGCCTTACGGTCACCGTAACCAGCAAATCAATATCACTGTGTGGCTT
CAGGCCGCCATCCACTGCGGAGCCGTACAAATGTACGGCCAGCAACGTCGGTTCGAGATGGCGCTCGATGACGCC
AACTACCTCTGATAGTTGAGTCGATACTTCGGCGATCACCGCTTCCCTCATGATGTTTAACTTTGTTTTAGGGCGAC
TGCCCTGCTGCGTAACATCGTTGCTGCTCCATAACATCAAACATCGACCCACGGCGTAACGCGCTTGCTGCTTGGA
TGCCCGAGGCATAGACTGTACCCCAAAAAAACAGTCATAACAAGCCATGAAAACCGCCACTGCGCCGTTACCACC
GCTGCGTTCGGTCAAGGTTCTGGACCAGTTGCGTGAGCGCATACGCTACTTGCATTACAGCTTACGAACCGAACAG
GCTTATGTCCACTGGGTTCGTGCCTTCATCCGTTTCCACGGTGTGCGTCACCCGGCAACCTTGGGCAGCAGCGAAG
TCGAGGCATTTCTGTCCTGGCTGGCGAACGAGCGCAAGGTTTCGGTCTCCACGCATCGTCAGGCATTGGCGGCCTT
GCTGTTCTTCTACGGCAAGGTGCTGTGCACGGATCTGCCCTGGCTTCAGGAGATCGGAAGACCTCGGCCGTCGCG
GCGCTTGCCGGTGGTGCTGACCCCGGATGAAGTGGTTCGCATCCTCGGTTTTCTGGAAGGCGAGCATCGTTTGTTC
GCCCAGCTTCTGTATGGAACGGGCATGCGGATCAGTGAGGGTTTGCAACTGCGGGTCAAGGATCTGGATTTCGAT
CACGGCACGATCATCGTGCGGGAGGGCAAGGGCTCCAAGGATCGGGCCTTGATGTTACCCGAGAGCTTGGCACC
CAGCCTGCGCGAGCAGGGGAATTAATTCCCACGGGTTTTGCTGCCCGCAAACGGGCTGTTCTGGTGTTGCTAGTTT
GTTATCAGAATCGCAGATCCGGCTTCAGGTTTGCCGGCTGAAAGCGCTATTTCTTCCAGAATTGCCATGATTTTTTC
CCCACGGGAGGCGTCACTGGCTCCCGTGTTGTCGGCAGCTTTGATTCGATAAGCAGCATCGCCTGTTTCAGGCTGT
CTATGTGTGACTGTTGAGCTGTAACAAGTTGTCTCAGGTGTTCAATTTCATGTTCTAGTTGCTTTGTTTTACTGGTTT
CACCTGTTCTATTAGGTGTTACATGCTGTTCATCTGTTACATTGTCGATCTGTTCATGGTGAACAGCTTTGAATGCAC
CAAAAACTCGTAAAAGCTCTGATGTATCTATCTTTTTTACACCGTTTTCATCTGTGCATATGGACAGTTTTCCCTTTG

ATATGTAACGGTGAACAGTTGTTCTACTTTTGTTTGTTAGTCTTGATGCTTCACTGATAGATACAAGAGCCATAAGA
ACCTCAGATCCTTCCGTATTTAGCCAGTATGTTCTCTAGTGTGGTTCGTTGTTTTTGCGTGAGCCATGAGAACGAAC
CATTGAGATCATACTTACTTTGCATGTCACTCAAAAATTTTGCCTCAAAACTGGTGAGCTGAATTTTTGCAGTTAAA
GCATCGTGTAGTGTTTTTCTTAGTCCGTTATGTAGGTAGGAATCTGATGTAATGGTTGTTGGTATTTTGTCACCATT
CATTTTTATCTGGTTGTTCTCAAGTTCGGTTACGAGATCCATTTGTCTATCTAGTTCAACTTGGAAAATCAACGTATC
AGTCGGGCGGCCTCGCTTATCAACCACCAATTTCATATTGCTGTAAGTGTTTAAATCTTTACTTATTGGTTTCAAAAC
CCATTGGTTAAGCCTTTTAAACTCATGGTAGTTATTTTCAAGCATTAACATGAACTTAAATTCATCAAGGCTAATCTC
TATATTTGCCTTGTGAGTTTTCTTTTGTGTTAGTTCTTTTAATAACCACTCATAAATCCTCATAGAGTATTTGTTTTCA
AAAGACTTAACATGTTCCAGATTATATTTTATGAATTTTTTTAACTGGAAAAGATAAGGCAATATCTCTTCACTAAA
AACTAATTCTAATTTTTCGCTTGAGAACTTGGCATAGTTTGTCCACTGGAAAATCTCAAAGCCTTTAACCAAAGGAT
TCCTGATTTCCACAGTTCTCGTCATCAGCTCTCTGGTTGCTTTAGCTAATACACCATAAGCATTTTCCCTACTGATGT
TCATCATCTGAGCGTATTGGTTATAAGTGAACGATACCGTCCGTTCTTTCCTTGTAGGGTTTTCAATCGTGGGGTTG
AGTAGTGCCACACAGCATAAAATTAGCTTGGTTTCATGCTCCGTTAAGTCATAGCGACTAATCGCTAGTTCATTTGC
TTTGAAAACAACTAATTCAGACATACATCTCAATTGGTCTAGGTGATTTTAATCACTATACCAATTGAGATGGGCTA
GTCAATGATAATTACTAGTCCTTTTCCTTTGAGTTGTGGGTATCTGTAAATTCTGCTAGACCTTTGCTGGAAAACTT
GTAAATTCTGCTAGACCCTCTGTAAATTCCGCTAGACCTTTGTGTGTTTTTTTTGTTTATATTCAAGTGGTTATAATTT
ATAGAATAAAGAAAGAATAAAAAAAGATAAAAAGAATAGATCCCAGCCCTGTGTATAACTCACTACTTTAGTCAGT
TCCGCAGTATTACAAAAGGATGTCGCAAACGCTGTTTGCTCCTCTACAAAACAGACCTTAAAACCCTAAAGGCTTA
AGTAGCACCCTCGCAAGCTCGGGCAAATCGCTGAATATTCCTTTTGTCTCCGACCATCAGGCACCTGAGTCGCTGT
CTTTTTCGTGACATTCAGTTCGCTGCGCTCACGGCTCTGGCAGTGAATGGGGGTAAATGGCACTACAGGCGCCTTT
TATGGATTCATGCAAGGAAACTACCCATAATACAAGAAAAGCCCGTCACGGGCTTCTCAGGGCGTTTTATGGCGG
GTCTGCTATGTGGTGCTATCTGACTTTTTGCTGTTCAGCAGTTCCTGCCCTCTGATTTTCCAGTCTGACCACTTCGGA
TTATCCCGTGACAGGTCATTCAGACTGGCTAATGCACCCAGTAAGGCAGCGGTATCATCAACAGGCTTACCCGTCT
TACTGTCGGGAATTCCCCGATAGAAAGCGTGAGAAACAGCGTACAGACGATTTAGAGATGTAGAGGTACTTTTAT
GCCGAGAAAACTTTTTGCGTGTGACAGTCCTTAAAATATACTTAGAGCGTAAGCGAAAGTAGTAGCGACAGCTATT
AACTTTCGGTTGCAAAGCTCTAGGATTTTTAATGGACGCAGCGCATCACACGCAAAAAGGAAATTGGAATAAATG
CGAAATTTGAGATGTTAATTAAAGACCTTTTTGAGGTCTTTTTTTCTTAGATTTTTGGGGTTATTTAGGGGAGAAAA
CATAGGGGGGTACTACGACCTCCCCCCTAGGTGTCCATTGTCCATTGTCCAAACAAATAAATAAATATTGGGTTTTT
AATGTTAAAAGGTTGTTTTTTATGTTAAAGTGAAAAAAACAGATGTTGGGAGGTACAGTGATGGTTGTAGATAGA
AAAGAAGAGAAAAAAGTTGCTGTTACTTTAAGACTTACAACAGAAGAAAATGAGATATTAAATAGAATCAAAGAA
AAATATAATATTAGCAAATCAGATGCAACCGGTATTCTAATAAAAAAATATGCAAAGGAGGAATACGGTGCATTTT
AAACAAAAAAAGATAGACAGCACTGGCATGCTGCCTATCTATGACTAAATTTTGTTAAGTGTATTAGCACCGTTATT
ATATCATGAGCGAAAATGTAATAAAAGAAACTGAAAACAAGAAAAATTCAAGAGGACGTAATTGGACATTTGTTT
TATATCCAGAATCAGCAAAAGCCGAGTGGTTAGAGTATTTAAAAGAGTTACACATTCAATTTGTAGTGTCTCCATT
ACATGATAGGGATACTGATACAGAAGGTAGGATGAAAAAAGAGCATTATCATATTCTAGTGATGTATGAGGGTAA
TAAATCTTATGAACAGATAAAAATAATTAACAGAAGAATTGAATGCGACTATTCCGCAGATTGCAGGAAGTGTGA
AAGGTCTTGTGAGATATATGCTTCACATGGACGATCCTAATAAATTTAAATATCAAAAAGAAGATATGATAGTTTA
TGGCGGTGTAGATGTTGATGAATTATTAAAGAAAACAACAACAGATAGATATAAATTAATTAAAGAAATGATTGA
GTTTATTGATGAACAAGGAATCGTAGAATTTAAGAGTTTAATGGATTATGCAATGAAGTTTAAATTTGATGATTGG
TTCCCGCTTTTATGTGATAACTCGGCGTATGTTATTCAAGAATATATAAAATCAAATCGGTATAAATCTGACCGATA
GATTTTGAATTTAGGTGTCACAAGACACTCTTTTTTCGCACCAGCGAAAACTGGTTTAAGCCGACTGCGCAAAAGA
CATAATCGATTCACAAAAAATAGGCACACGAAAAACAAGTTAAGGGATGCAGTTTATGCATCCCTTAACTTACTTA
TTAAATAATTTATAGCTATTGAAAAGAGATAAGAATTGTTCAAAGCTAATATTGTTTAAATCGTCAATTCCTGCATG
TTTTAAGGAATTGTTAAATTGATTTTTTGTAAATATTTTCTTGTATTCTTTGTTAACCCATTTCATAACGAAATAATTA

TACTTTTGTTTATCTTTGTGTGATATTCTTGATTTTTTTCTACTTAATCTGATAAGTGAGCTATTCACTTTAGGTTTAG
GATGAAAATATTCTCTTGGAACCATACTTAATATAGAAATATCAACTTCTGCCATTAAAAGTAATGCCAATGAGCGT
TTTGTATTTAATAATCTTTTAGCAAACCCGTATTCCACGATTAAATAAATCTCATTAGCTATACTATCAAAAACAATT
TTGCGTATTATATCCGTACTTATGTTATAAGGTATATTACCATATATTTTATAGGATTGGTTTTTAGGAAATTTAAAC
TGCAATATATCCTTGTTTAAAACTTGGAAATTATCGTGATCAACAAGTTTATTTTCTGTAGTTTTGCATAATTTATGG
TCTATTTCAATGGCAGTTACGAAATTACACCTCTTTACTAATTCAAGGGTAAAATGGCCTTTTCCTGAGCCGATTTCA
AAGATATTATCATGTTCATTTAATCTTATATTTGTCATTATTTTATCTATATTATGTTTTGAAGTAATAAAGTTTTGAC
TGTGTTTTATATTTTTCTCGTTCATTATAACCCTCTTTAATTTGGTTATATGAATTTTGCTTATTAACGATTCATTATAA
CCACTTATTTTTTGTTTGGTTGATAATGAACTGTGCTGATTACAAAAATACTAAAAATGCCCATATTTTTTCCTCCTTA
TAAAATTAGTATAATTATAGCACGAGCTCTGATAAATATGAACATGATGAGTGATCGTTAAATTTATACTGCAATCG
GATGCGATTATTGAATAAAAGATATGAGAGATTTATCTAATTTCTTTTTTCTTGTAAAAAAAGAAAGTTCTTAAAGG
TTTTATAGTTTTGGTCGTAGAGCACACGGTTTAACGACTTAATTACGAAGTAAATAAGTCTAGTGTGCCCACAGGTT
GGCTGATAAGTCCCCGGTCTTGatgGCGGATAAACAAAAAAACGGTATTAAAGCAAACTTCAAGATCCGGCATAAT
ATTGAAGATGGGGGTGTCCAGCTCGCCGACCACTACCAACAAAACACTCCGATAGGTGACGGGCCAGTGTTACTG
CCGGACAATCATTACCTTAGCTACCAAAGCAAGTTGAGCAAGGACCCTAATGAGAAACGGGATCACATGGTACTC
TTGGAGTTTGTTACCGCGGCTGGGATAACGCTCGGTATGGATGAACTTTACAAGGGAGACCGGGGACTTATCAGC
CAACCTGTGGGGATCCTACACTTGCTGCTAATGCGCTCGTTGAAAACGGAGCGAAAGAAGTATATGCATGCTGTA
CACACCCTGTACTATCAGGCCCTGCGGTTGAACGGATTAATAATTCAACAATTAAAGAGCTTGTTGTGACAAACAG
CATCAAGCTTCCTGAAGAAAAGAAAATTGAACGCTTTAAGCAGCTTTCAGTCGGACCGCTTCTGGCCGAAGCGATT
ATTCGCGTTCATGAGCAGCAATCAGTCAGCTATCTGTTCAGCTAAACCATTTTTCGAGGTTTAAATCCTTATCGTTAT
GGGTATTGTTTGTAATAGGACAACTAAAACGACAAGAGGTCGACTAAGGAGGTGAGAGCAATGGAAAAAAAGGA
ATTTCGTGTTTTGATAAAATACTGTTTTCTGAAGGGAAAAAATACAGTGGAAGCAAAAACTTGGCTTGATAATGAG
TTTCCGGACTCTGCCCCAGGGAAATCAACAATAATTGATTGGTATGCAAAATTCAAGCGTGGTGAAATGAGCACG
GAGGACGGTGAACGCAGTGGACGCCCGAAAGAGGTGGTTACCGACGAAAACATCAAAAAAATCCACAAAATGAT
TTTGAATGACCGTAAAATGAAGTTGATCGAGATAGCAGAGGCCTTAAAGATATCAAAGGAACGTGTTGGTCATAT
CATTCATCAATATTTGGATATGCGGAAGCTCTGTGCGAAATGGGTGCCGCGCGAGCTCACATTTGACCAAAAACAA
CGACGTGTTGATGATTCTAAGCGGTGTTTGCAGCTGTTAACTCGTAATACACCCGAGTTTTTCCGTCGATATGTGAC
AATGGATGAAACATGGCTCCATCACTACACTCCTGAGTCCAATCGACAGTCGGCTGAGTGGACAGCGACCGGTGA
ACCGTCTCCGAAGCGTGGAAAGACTCAAAAGTCCGCTGGCAAAGTAATGGCCTCTGTTTTTTGGGATGCGCATGG
AATAATTTTTATCGATTATCTTGAGAAGGGAAAAACCATCAACAGTGACTATTATATGGCGTTATTGGAGCGTTTG
AAGGTCGAAATCGCGGCAAAACGGCCCCACATGAAGAAGAAAAAAGTGTTGTTCCACCAAGACAACGCACCGTG
CCACAAGTCATTGAGAACGATGGCAAAAATTCATGAATTGGGCTTCGAATTGCTTCCCCACCCGCCGTATTCTCCA
GATCTGGCCCCCAGCGACTTTTTCTTGTTCTCAGACCTCAAAAGGATGCTCGCAGGGAAAAAATTTGGCTGCAATG
AAGAGGTGATCGCCGAAACTGAGGCCTATTTTGAGGCAAAACCGAAGGAGTACTACCAAAATGGTATCAAAAAAT
TGGAAGGTCGTTATAATCGTTGTATCGCTCTTGAAGGGAACTATGTTGAATAATAAAGGGGATCCGTCGACCTGCA
GCCAAGCTT-3’

SUPPLEMENTAL FIGURE LEGENDS

Figure S1. Cartoon diagrams of the Tn*FLXopen3* series. A) Sequence of unmodified Tn*FLXopen3* inserted in frame in theoretical gene introduces three stop codons prior to the reporter. Orange is the theoretical sequence in which the transposon was inserted. Bent arrow indicates a promoter, and stop sign indicates a stop codon. Red is the sequence of the ITR, green is the sequence of the reporter, and blue are bases added to preserve the open reading frame. The TA sequence targeted for integration is bolded. Letters above the sequence line indicate the translated amino acid where X is theoretically any residue. B) Sequences of 10 different modified Tn*FLXopen3* transposons to remove in frame stop codons. In white are nucleotide alterations made to preserve the reading frame through the ITR. In the last two sequences a single base pair was inserted (blue) to move the subsequent stop codons out of frame. None of the resulting transposons exhibited appreciable transposition frequency and were discarded.

Figure S2. Three dimensional modeling of mNeongreen-FtsZ fusion proteins. The Tn*FLXopenmNeongreen* (green) FtsZ fusion (pink) structures were modeled using the ColabFold (1) software, which is based on the structure prediction provided by alphafold2 (2) and super-imposed onto the *B. subtilis* monomer (PDB 2VAM) (3).

Figure S3. Micrographs of original Tn*FLXopenmVenusC155* isolate strains. Fluorescence micrographs of Tn*FLXopenmVenusC155* fusion candidates in a strain expressing DivIVA-mVenusN154 at the native locus. While each strain may have contained multiple transposon insertions, the strains are annotated according to what would eventually be a linked candidate yielding fluorescence when expressed from an IPTG-inducible promoter in **Fig 4B**. Membrane stained with FM4-64 (false colored in red) and mVenus fluorescence (false colored in green). The following strains were used to generate each panel: *ahpC* (DK6013), *divIVA* (DK6012), *ezrA* (DK7191), *glnA* (DK7197), *msmX* (DK7196), *rocD* (DK6008), and *ybbC* (DK6035). Bar represents 5 µm.

SUPPLEMENTAL REFERENCES

1 Mirdita M, Ovchinnikov S and Steinegger M. 2021. ColabFold - Making protein folding accessible to all. bioRxiv, doi: 10.1101/2021.08.15.456425

2 Jumper, J., Evans, R., Pritzel, A. *et al.* 2021. Highly accurate protein structure prediction with AlphaFold. Nature **596,** 583–589.

3. Oliva MA, Trambaiolo, Loewe J. 2007 Structural Insights into the Conformational Variability of FtsZ. JMB **373**, 1229-1242

SUPPLEMENTAL FIGURES

Figure S1.

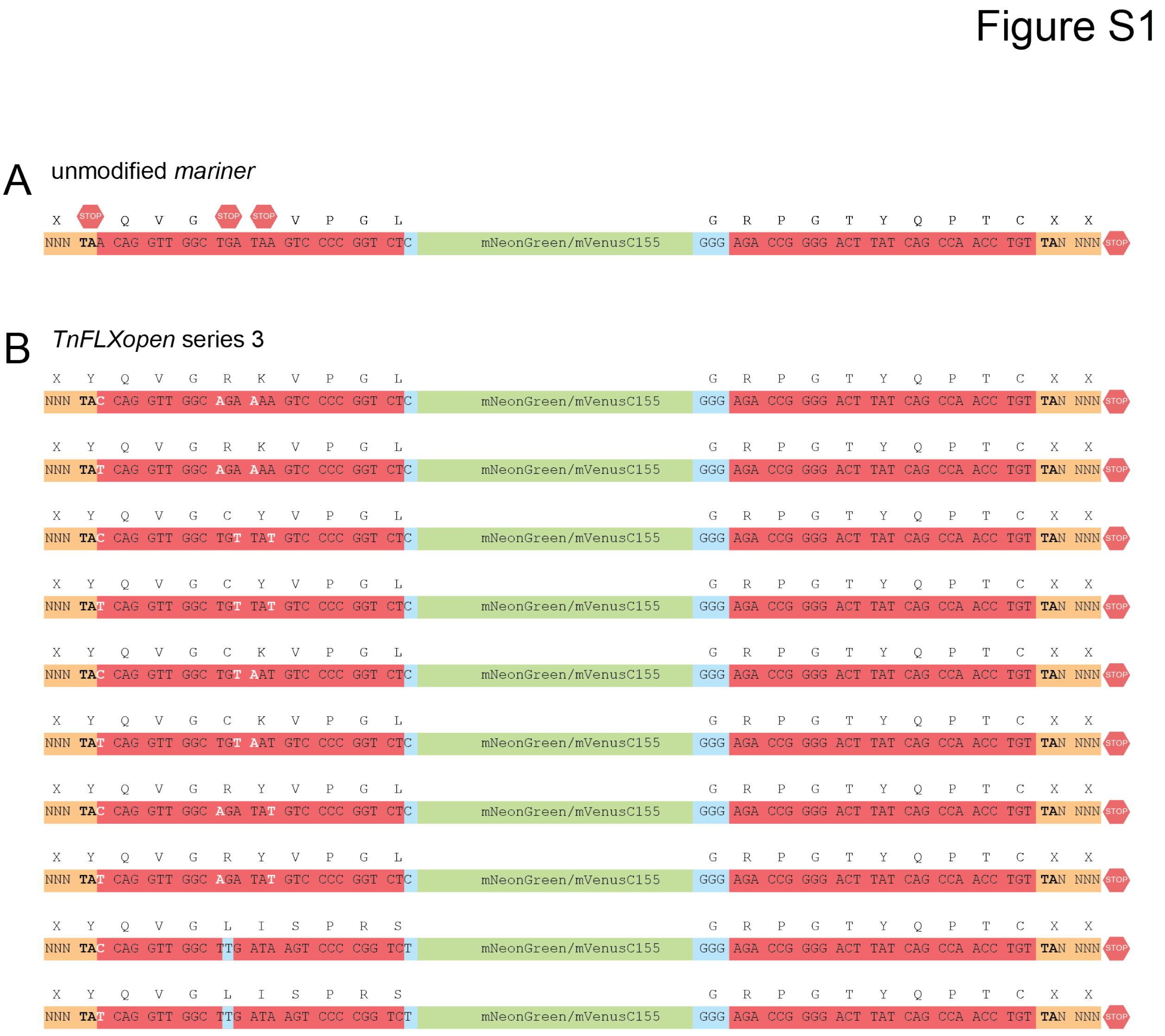

Figure S2

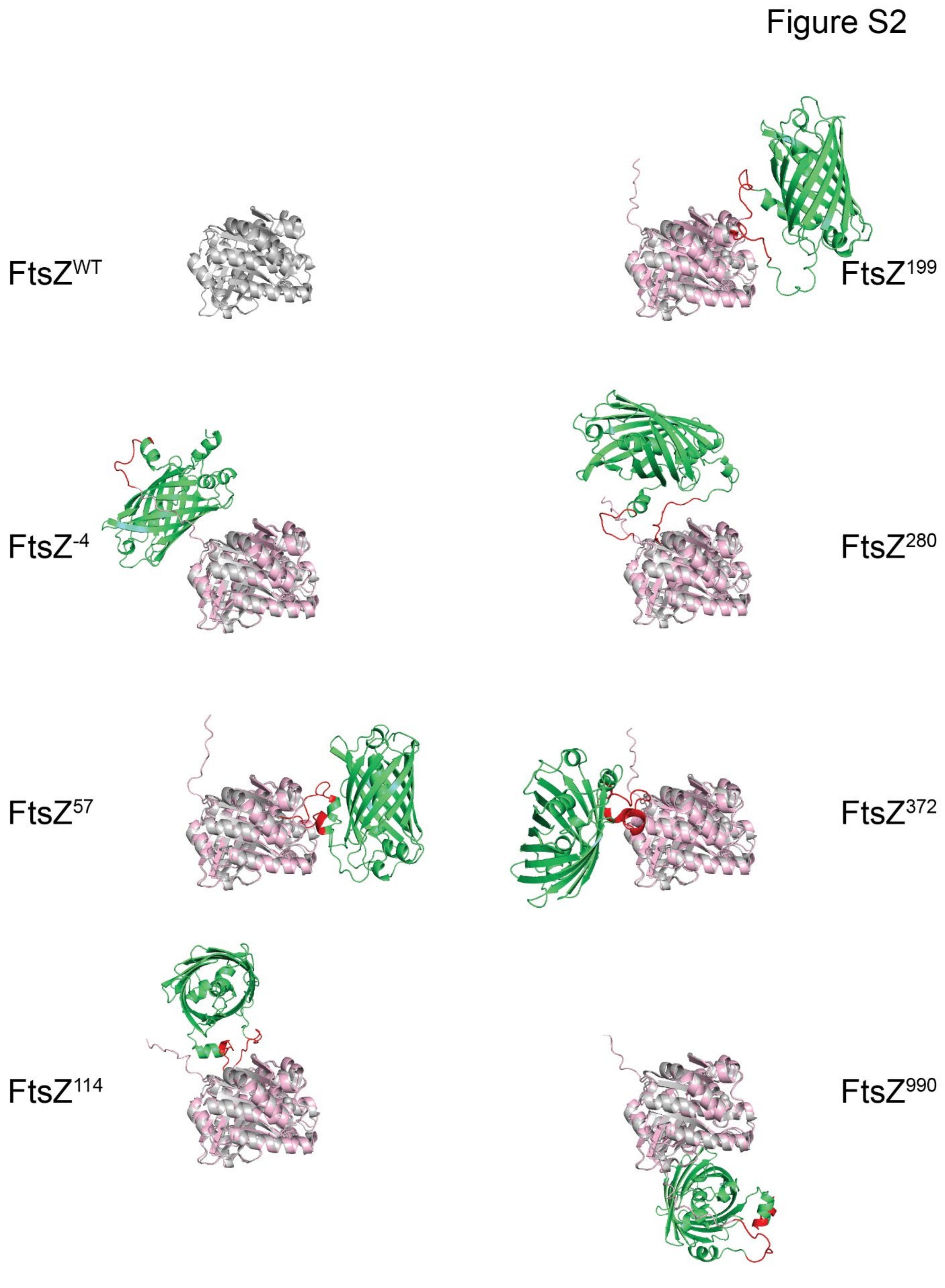
Figure S2
FtsZ^{WT}
FtsZ^{199}
FtsZ^{-4}
FtsZ^{280}
FtsZ^{57}
FtsZ^{372}
FtsZ^{114}
FtsZ^{990}

Figure S3

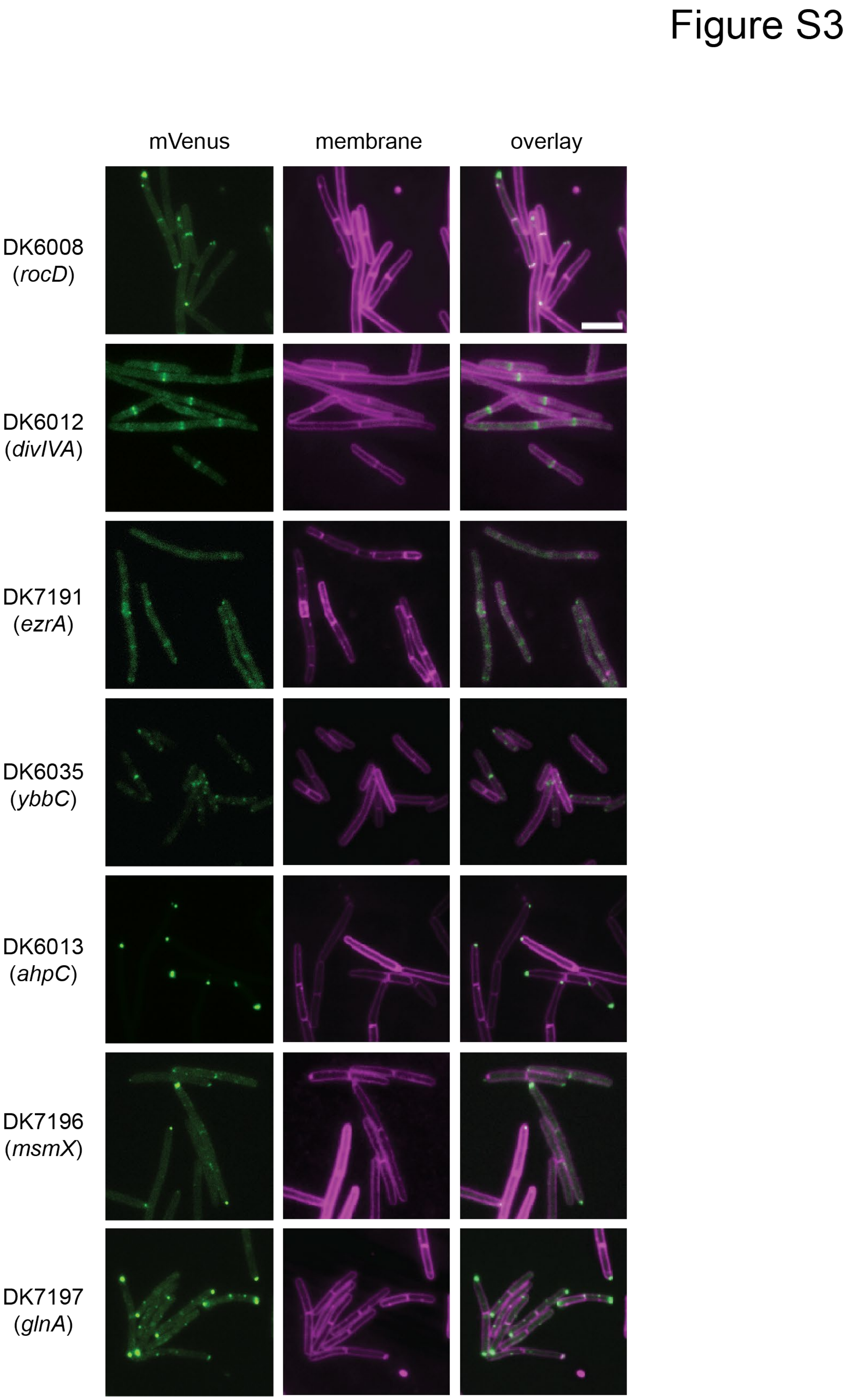
Figure S3
mVenus
membrane
overlay
DK6008
(*rocD*)
DK6012
(*divIVA*)
DK7191
(*ezrA*)
DK6035
(*ybbC*)
DK6013
(*ahpC*)
DK7196
(*msmX*)
DK7197
(*glnA*)
